# Tat-NTS peptide protects neurons against cerebral ischemia-reperfusion injury via ANXA1 SUMOylation in microglia

**DOI:** 10.7150/thno.85390

**Published:** 2023-10-16

**Authors:** Huijuan Zhou, Lulu Yan, Hezhou Huang, Xing Li, Qian Xia, Lu Zheng, Bin Shao, Qian Gao, Ning Sun, Jing Shi

**Affiliations:** 1 Department of Neurobiology, School of Basic Medicine, Tongji Medical College, Huazhong University of Science and Technology, Wuhan, Hubei 430030, China.; 2 The Institute for Brain Research, Collaborative Innovation Center for Brain Science, Huazhong University of Science and Technology, Wuhan, Hubei 430030, China.; 3 Key Laboratory of Neurological Diseases, Ministry of Education, Wuhan, Hubei 430030, China.; 4 Department of Anesthesiology, Tongji Hospital, Tongji Medical College, Huazhong University of Science and Technology, Wuhan, Hubei 430030, China.; 5 Department of Clinical Laboratory, The First Affiliated Hospital of Zhengzhou University, Zhengzhou, Henan 450052, China.; 6 Department of Pathophysiology, School of Basic Medicine, Tongji Medical College, Huazhong University of Science and Technology, Wuhan, Hubei 430030, China.

**Keywords:** Tat-NTS peptide, Annexin-A1, cerebral ischemia-reperfusion injury, microglia, SUMOylation

## Abstract

**Rationale:** Recent studies indicate that microglial activation and the resulting inflammatory response could be potential targets of adjuvant therapy for ischemic stroke. Many studies have emphasized a well-established function of Annexin-A1 (ANXA1) in the immune system, including the regulation of microglial activation. Nevertheless, few therapeutic interventions targeting ANXA1 in microglia for ischemic stroke have been conducted. In the present study, Tat-NTS, a small peptide developed to prevent ANXA1 from entering the nucleus, was utilized. We discovered the underlying mechanism that Tat-NTS peptide targets microglial ANXA1 to protect against ischemic brain injury.

**Methods:** Preclinical studies of ischemic stroke were performed using an oxygen-glucose deprivation and reperfusion (OGD/R) cell model in vitro and the middle cerebral artery occlusion (MCAO) animal model of ischemic stroke in vivo. Confocal imaging and 3D reconstruction analyses for detecting the protein expression and subcellular localization of microglia in vivo. Co-immunoprecipitation (Co-IP), immunoblotting, ELISA, quantitative real-time PCR (qRT-PCR), Luciferase reporter assay for determining the precise molecular mechanism. Measurement on the cytotoxicity of Tat-NTS peptide for microglia was assessed by CCK-8 and LDH assay. TUNEL staining was used to detect the microglia conditioned medium-mediated neuronal apoptosis. Adeno-associated viruses (AAVs) were injected into the cerebral cortex, striatum and hippocampal CA1 region of adult male Cx3cr1-Cre mice, to further verify the neurofunctional outcome and mechanism of Tat-NTS peptide by TTC staining, the modified Neurological Severity Score (mNSS) test, the open field test (OFT), the novel object recognition task (NORT), the Morris water maze (MWM) test, the long-term potentiation (LTP) and the Transmission electron microscopy (TEM).

**Results:** It was observed that administration of Tat-NTS led to a shift of subcellular localization of ANXA1 in microglia from the nucleus to the cytoplasm in response to ischemic injury. Notably, this shift was accompanied by an increase in ANXA1 SUMOylation in microglia and a transformation of microglia towards an anti-inflammatory phenotype. We confirmed that Tat-NTS-induced ANXA1 SUMOylation in microglia mediated IKKα degradation via NBR1-dependent selective autophagy, then blocking the activation of the NF-κB pathway. As a result, the expression and release of the pro-inflammatory factors IL-1β and TNF-α were reduced in both in vitro and in vivo experiments. Furthermore, we found that Tat-NTS peptide's protective effect on microglia relieved ischemic neuron apoptosis. Finally, we demonstrated that Tat-NTS peptide administration, through induction of ANXA1 SUMOylation in microglia, reduced infarct volume, improved neurological function and facilitated behavioral recovery in MCAO mice.

**Conclusions:** Our study provides evidence for a novel mechanism of Tat-NTS peptide in regulating microglial ANXA1 function and its substantial neuroprotective effect on neurons with ischemic injuries. These findings suggest that Tat-NTS peptides have a high potential for clinical application and may be a promising therapeutic candidate for treating cerebral ischemia.

## Introduction

Ischemic stroke is a principal cause of devastating disability and death in patients with cerebrovascular disease 1. Currently, tPA is the only approved pharmacological therapy proven to be effective in acute ischemic stroke 2. Unfortunately, the clinical use of tPA is limited due to its narrow therapeutic window of efficacy (within 4.5 h after ischemic stroke) and the risk of intracerebral hemorrhage 3, 4. tPA should only be used in < 5% of patients with acute ischemic stroke. In addition, nearly 17% to 34% of ischemic stroke patients experience early arterial reocclusion and poor long-term prognosis after tPA use 5. Taken together, these limitations highlight the urgent need for exploring novel adjuvant therapies that can effectively improve the limited success rate of stroke reperfusion therapy.

Microglia act as key regulators of inflammatory responses within the central nervous system (CNS) and communicate with other types of nerve cells 6, 7, which is essential for maintaining CNS homeostasis 8. The microglial response is the first step before the innate immune response triggered after stroke 9. Based on different functional states in response to environmental stimuli, microglia are activated towards different phenotypes, which are often divided into two broad categories: pro-inflammatory (classically activated) and anti-inflammatory (alternatively activated) phenotypes; this process is referred to as microglial polarization 10, 11. Pro-inflammatory microglia, by secreting cytokines such as interleukin-1β (IL-1β), interleukin-6 (IL-6), tumor necrosis factor-α (TNF-α), inducible nitric oxide synthase (iNOS), and Fc gamma RII/RIII (CD16/32), induce further inflammation and accelerate the pathological course of cerebral ischemia. In contrast, anti-inflammatory microglia initiate the amelioration of brain injury after ischemic stroke by secreting key anti-inflammatory mediators such as arginase-1 (Arg-1), transforming growth factor-β (TGF-β), interleukin-4 (IL-4), interleukin-10 (IL-10), and CD206 12.

Numerous recent studies have confirmed that altering the activation state of microglia can attenuate ischemic injury 7, 12, 13. For example, ANXA1 regulates microglial polarization through the FPR2/ALX-stimulated AMPK/mTOR pathway to protect against cerebral ischemia-reperfusion injury 13; PARP14 suppresses microglial activation and further promotes post-stroke functional recovery through LPAR5 7. ANXA1, a member of the annexin protein superfamily that mimics the effects of glucocorticoids 14. A number of studies have highlighted a well-established role for ANXA1 in the immune system, including regulation of microglial activation, endothelial integrity and leukocyte trafficking 15, 16. In transient ischemic attack, nuclear accumulation of ANXA1 in microglia was associated with microglial polarization 17. Furthermore, overexpressing ATP-binding cassette transporter A1 (ABCA1) recruited and confined ANXA1 to the cytoplasm and blocked nuclear translocation of ANXA1, which improved retinal ganglion cell survival in the mouse model of ischemia-reperfusion 18. Cytoplasmic AXNA1 modified by SUMOylation shifted microglia towards an anti-inflammatory phenotype after ischemia-reperfusion injury 12. Thus, our study positions microglial ANXA1 as a therapeutic target against cerebral ischemia and may achieve a multiplier effect.

In this study, we reported the nuclear translocation of ANXA1 in microglia after cerebral ischemic-reperfusion injury in vivo, then used a Tat-NTS peptide, a cell-penetrating peptide obtained by binding the transcriptional transactivator (Tat) domain to the unique nuclear translocation signal (NTS) sequence of ANXA1 19, to block the nuclear translocation of ANXA1 in microglia and evaluated the neuroprotective potential of Tat-NTS peptide against ischemic injury. Tat-NTS peptide could transform ANXA1 from nuclear localization to cytoplasmic localization after cerebral ischemic-reperfusion injury, modulate ischemic brain injury-induced microglial polarization towards an anti-inflammatory phenotype and attenuate microglial immune response. Furthermore, we manipulated gene overexpression in microglia/macrophages from specific regions of the model mouse brain and clarified the precise molecular mechanism that Tat-NTS peptide facilitates NBR1-mediated selective autophagic degradation of IκB kinase α (IKKα) to limit cerebral ischemic-reperfusion injury-mediated NF-κB pathway activation, and this process is ANXA1 SUMOylation-dependent. Finally, we quantified the infarct volume, neurological damage, cognitive function, motor coordination, synaptic plasticity, and levels of autophagy, inflammation, and apoptosis in MCAO/R model mice treated with Tat-NTS peptide, which provided conclusive evidence that Tat-NTS peptide regulated ANXA1 SUMOylation in microglia/macrophages and exerted a robust neuroprotective effect against cerebral ischemia/reperfusion injury.

## Materials and Methods

### Animals

**Figure 1:**
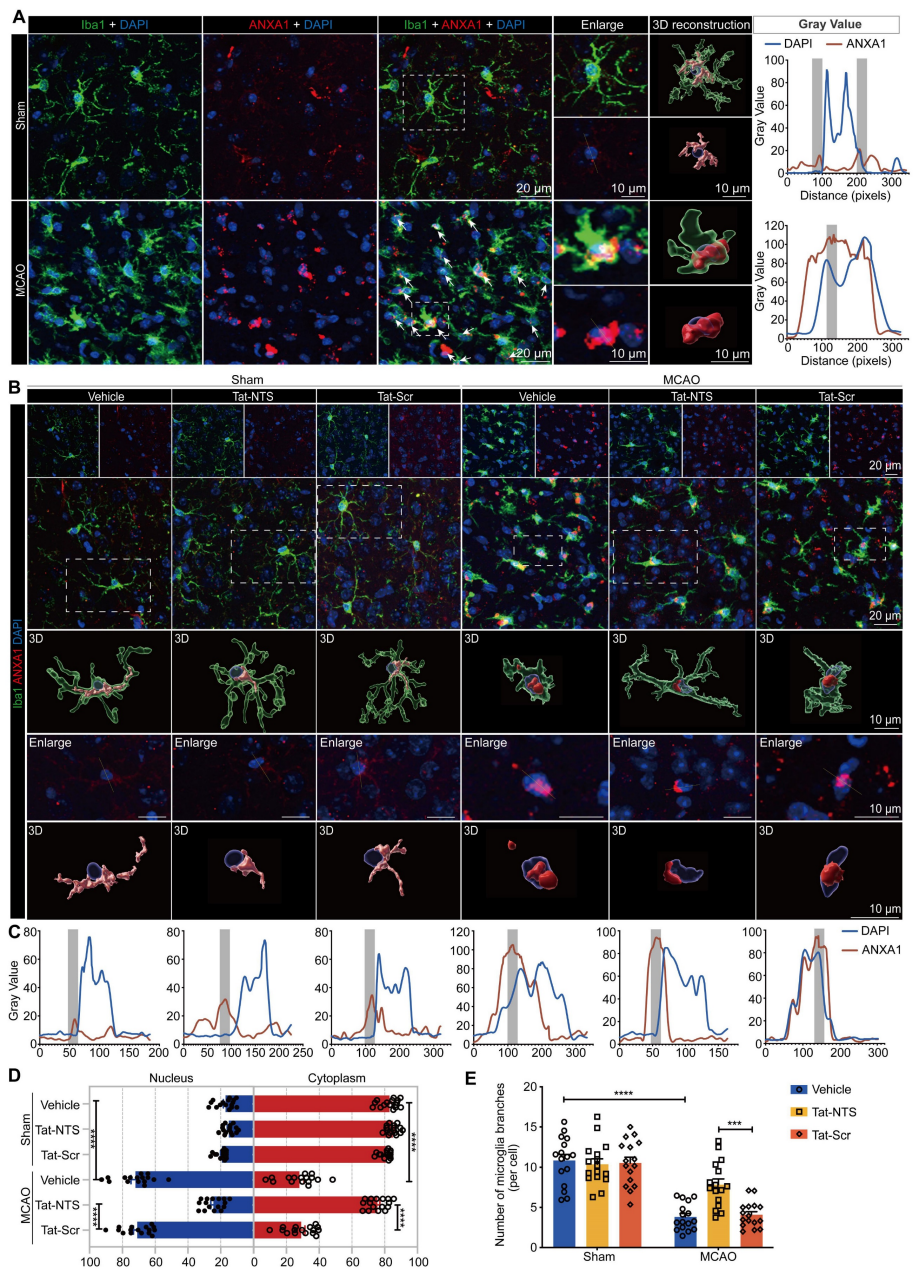
** Tat-NTS peptide shifts the subcellular localization of ANXA1 in microglia from the nucleus to the cytoplasm after ischemic injury. (A)** Representative immunofluorescence images of triple labeling of ANXA1 (red), Iba1 (cell-specific marker for microglia, green) and DAPI (blue) from ischemic penumbra of brain tissue of wild-type mice after MCAO surgery. Column 3, merged ANXA1, Iba1 and DAPI fluorescence, white arrows indicate cells with ANXA1-positive nuclei, scale bar of columns 1-3, 20 μm; Column 4, enlargement images of areas of interest indicated by white dashed box in column 3, upper section shows triple fluorescent labeling, lower section shows double labeling of ANXA1 and DAPI to visually demonstrate the subcellular localization of ANXA1 relative to the nucleus; Column 5, 3D reconstruction of individual microglia in the corresponding sections of column 4, ANXA1 (gray value below 40, light red; gray value above 40, dark red), Iba1 (translucent green), DAPI (transparent blue), the scale bar of columns 4-5, 10 μm; Column 6, merged profiles of gray value of ANXA1 (red line) and DAPI (blue line) signals along the yellow lines crossing microglia as shown in the lower section of column 4, gray value was determined by Fiji ImageJ software. Light gray areas indicate the ANXA1 peak. **(B)** Representative immunofluorescence images of triple labeling of ANXA1 (red), Iba1 (green), and DAPI (blue) from the ischemic penumbra of wild-type mouse brain tissue, showing the changes in microglia and ANXA1 under Tat-NTS peptide treatment after ischemic injury. Row 2, merge of ANXA1, Iba1, and DAPI fluorescence, scale bar of row 1-2, 20 μm; Row 3-5, enlargement images and 3D reconstruction for individual microglia in areas of interest indicated by white dashed box in row 2, presentation of details was the same as in A, scale bar, 10 μm; **(C)** Merged profiles of gray value of ANXA1 (red line) and DAPI (blue line) signals along the yellow lines crossing microglia as shown in row 4 of B, gray value was determined by Fiji ImageJ software. Light gray areas indicate the ANXA1 peak. **(D)** Quantitative analysis of the percentage of nucleocytoplasmic fluorescence distribution of ANXA1 using Fiji ImageJ software. **(E)** Number of microglial branches. Each data point in D and E represents the average of all microglia in a single field of view, 16 randomly selected fields of view from n = 4 mice. Data are presented as means ± SEM and analyzed by one-way ANOVA (D) or two-way ANOVA (E) with Tukey's post hoc test. ***p < 0.001 and ****p < 0.0001.

**Figure 2:**
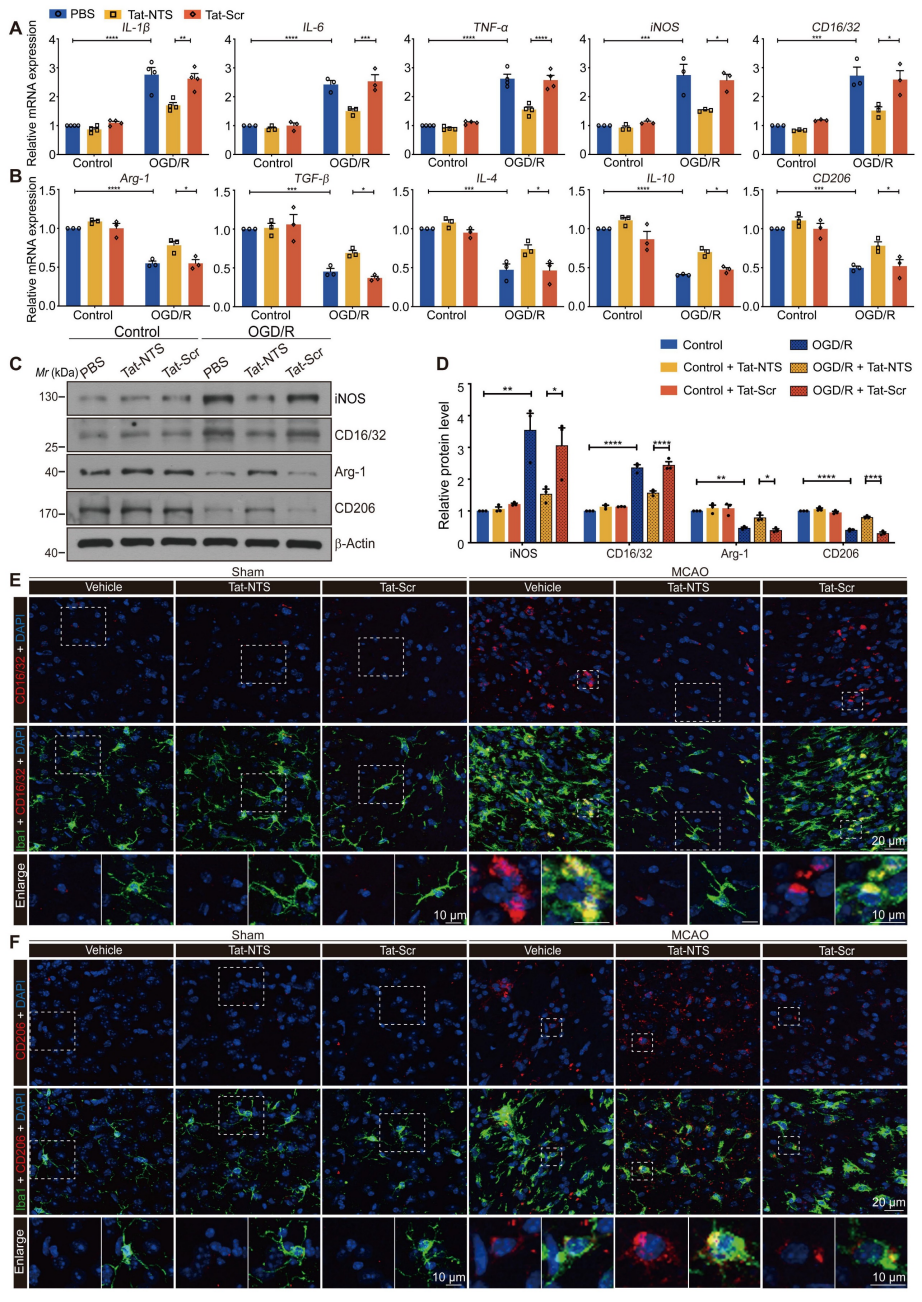
** Tat-NTS peptide promotes microglial transformation towards an anti-inflammatory phenotype after cerebral ischemia. (A and B)** qRT-PCR analysis was performed to measure the mRNA expression levels of pro-inflammatory marker genes (A) and anti-inflammatory marker genes (B) in primary cultured microglia under Tat-NTS peptide treatment.** (C)** Representative images of immunoblot analysis of iNOS, CD16/32, Arg-1 and CD206 in primary cultured microglia. Mr, relative molecular mass. **(D)** Quantitative analysis of the data shown in C. **(E)** Representative immunofluorescence images of triple labeling of CD16/32 (red), Iba1 (green), and DAPI (blue) and **(F)** Representative immunofluorescence images of triple labeling of CD206 (red), Iba1 (green), and DAPI (blue) from the ischemic penumbra of wild-type mice brain tissue; row 3, enlargement images of areas of interest indicated in row 1 and 2 by white dashed box; the scale bar of row 1-2, 20 μm; the scale bar of row 3, 10 μm. Data are presented as means ± SEM and analyzed by two-way ANOVA followed by Tukey's post hoc test for (A), (B) and (D). *p < 0.05, **p < 0.01, ***p < 0.001, and ****p < 0.0001.

**Figure 3:**
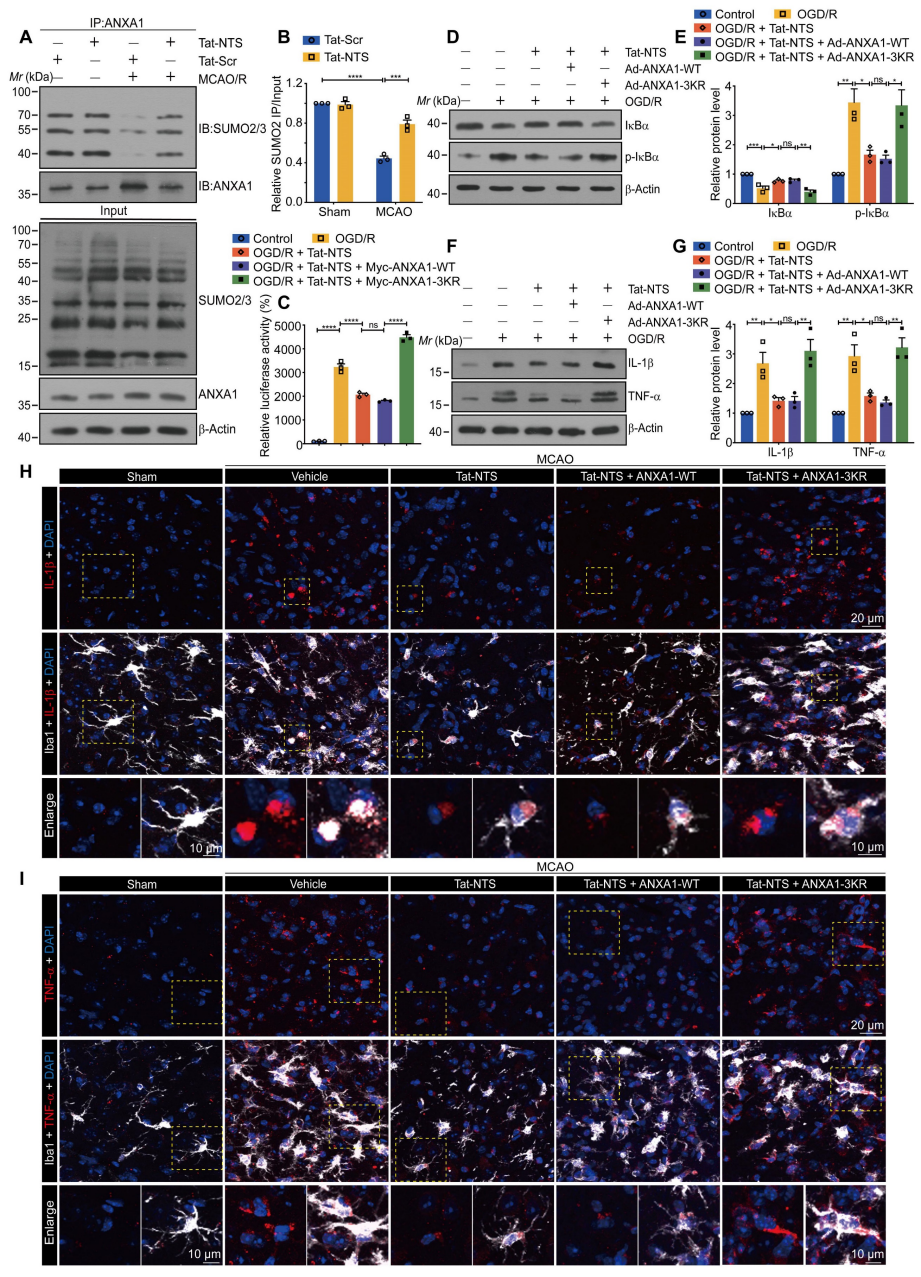
** Tat-NTS peptide induces an increase in ANXA1 SUMOylation and suppresses ischemic injury-induced NF-κB activity and the expression of pro-inflammatory factor IL-1β and TNF-α by ANXA1 SUMOylation. (A and B)** The interaction between endogenous ANXA1 and SUMO2 was detected in MCAO/R mice treated with Tat-NTS or Tat-Scr peptide by IP assay (A) and quantitative analysis (B). **(C)** The transcriptional activity of NF-κB p65 in HEK293T cells was detected by dual-luciferase reporter assay. **(D and E)** The representative immunoblotting for protein expression of IκBα and its phosphorylation level in microglial cells (D) and quantitative analysis (E).** (F)** Protein expression of IL-1β and TNF-α in microglia was analyzed by immunoblot analysis.** (G)** Quantitative analysis of the blots shown in F. **(H)** Representative immunofluorescence images of triple labeling of IL-1β (red), Iba1 (grey) 54 and DAPI (blue) and **(I)** Representative immunofluorescence images of triple labeling of TNF-α (red), Iba1 (grey) and DAPI (blue) from ischemic penumbra of brain tissue of Cx3cr1-Cre mice; row 3, enlargement images of areas of interest indicated in row 1 and 2 by yellow dashed box; the scale bar of row 1-2, 20 μm; the scale bar of row 3, 10 μm. Data are presented as means ± SEM and analyzed by one-way ANOVA (C, E and G) or two-way ANOVA (B) with Tukey's post hoc test. ns, not significant; p > 0.05; *p < 0.05; **p < 0.01; ***p < 0.001; and ****p < 0.0001.

**Figure 4:**
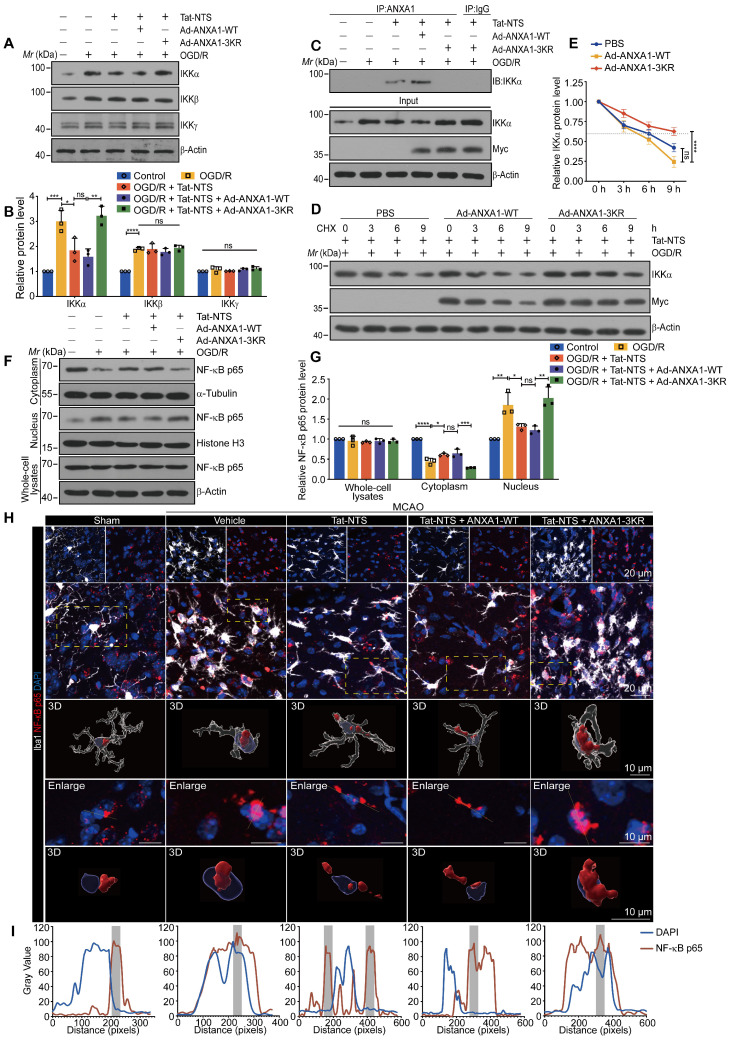
** Tat-NTS peptide promotes IKKα protein degradation by ANXA1 SUMOylation to suppress the ischemic injury-mediated NF-κB p65 nuclear translocation. (A)** Representative immunoblots of IKKα, IKKβ or IKKγ in microglia. **(B)** Quantitative analysis of the immunoblots in (A). **(C)** Interaction between ANXA1 and IKKα in primary cultured microglia was measured by Co-IP analysis.** (D and E)** Tat-NTS peptide shortens the half-life of endogenous IKKα in primary microglial cells. The protein level of IKKα was analyzed by immunoblotting (D) and quantitative analysis (E). **(F and G)** Representative immunoblotting for protein expression of NF-κB p65 in cytoplasmic extracts, nuclear extracts and whole cell lysates in primary cultured microglia (F) and quantitative analysis (G). **(H)** Representative immunofluorescence images of triple labeling of NF-κB p65 (red), Iba1 (grey) and DAPI (blue) from ischemic penumbra of brain tissue of Cx3cr1-Cre mice. Row 2, merge of NF-κB p65, Iba1 and DAPI fluorescence, the scale bar of row 1-2, 20 μm; row 3-5, enlargement images and 3D reconstruction for individual microglia in areas of interest indicated in the row by yellow dashed box, NF-κB p65 (deep red), Iba1(transparent gray) and DAPI (transparent blue) were shown in 3D reconstruction images, the scale bar of row 3-5, 10 μm; **(I)** Merged profiles of gray value of NF-κB p65 (red line) and DAPI (blue line) signals along the yellow lines crossing microglia as shown in row 4 of (H), gray value was determined by Fiji ImageJ software. Light gray areas indicate the NF-κB p65 peak. Data are presented as means ± SEM and analyzed by one-way ANOVA (B and G) or two-way ANOVA (E) with Tukey's post hoc test. ns, not significant; p > 0.05; *p < 0.05; **p < 0.01; ***p < 0.001; and ****p < 0.0001.

**Figure 5:**
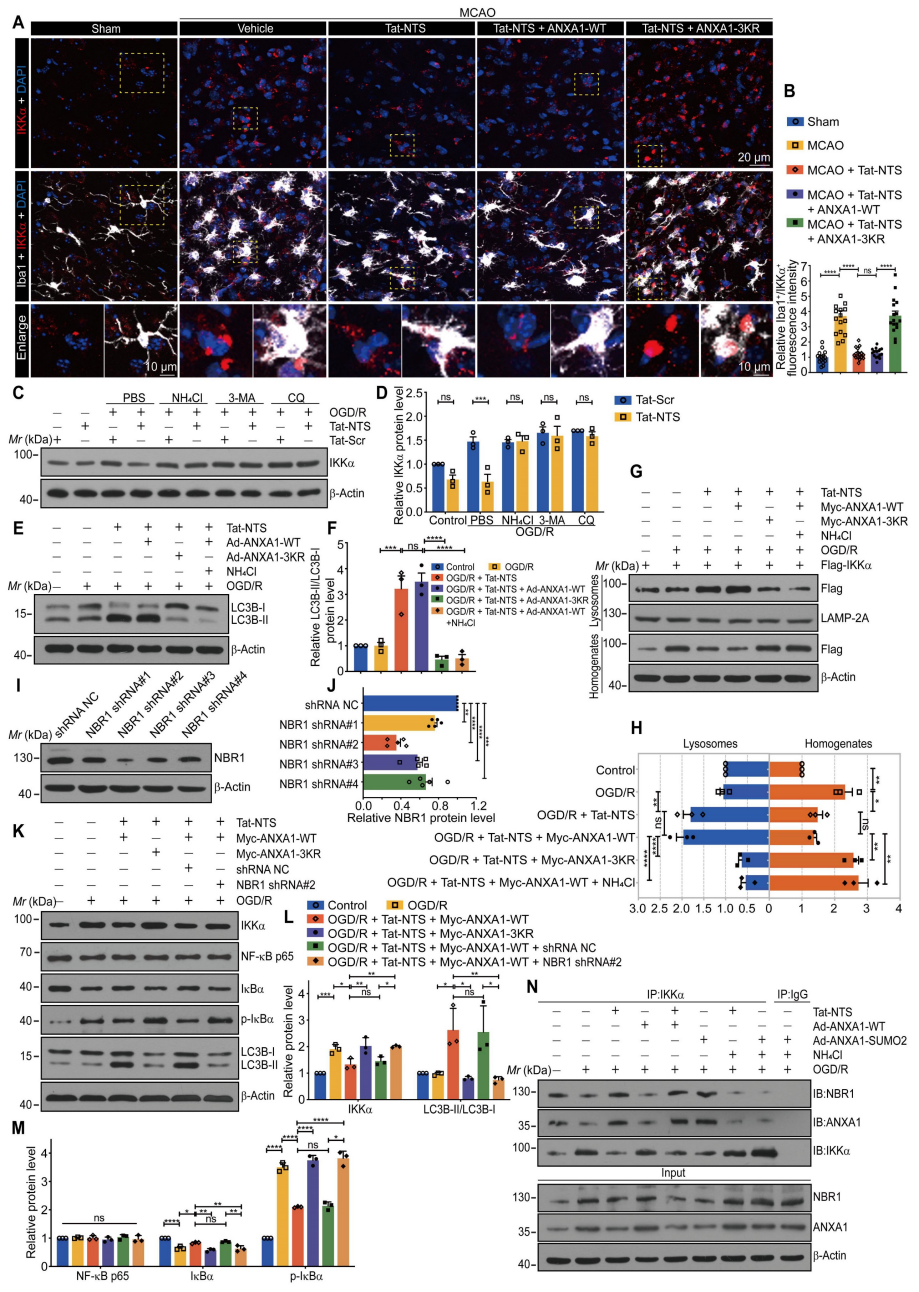
** Tat-NTS peptide promotes NBR1-dependent selective autophagic degradation of IKKα. (A)** Representative immunofluorescence images of triple labeling of IKKα (red), Iba1 (grey) and DAPI (blue) from ischemic penumbra of brain tissue of Cx3cr1-Cre mice; row 3, enlargement images of areas of interest indicated in row 1 and 2 by yellow dashed box; the scale bar of row 1-2, 20 μm; the scale bar of row 3, 10 μm. **(B)** Quantifcation of the IKKα^+^ fluorescence intensity in Iba1^+^ cells by Fiji imageJ. Each data point represents the average fluorescence intensity of all positive cells from a single field of view, 16 randomly selected fields of view from n = 4 mice. **(C)** Immunoblot analysis showing the protein expression of IKKα in microglia treated with Tat-NTS peptide or Tat-Scr peptide and NH_4_Cl, 3-MA or CQ. **(D)** Quantitative analysis of the immunoblots in (C). **(E)** Immunoblot analysis of LC3 protein expression in microglia. **(F)** Quantitative analysis of the immunoblots in (E). **(G)** Immunoblot analysis of the indicated proteins in HEK293T cell lines. **(H)** Quantitative analysis of the immunoblots in (G). **(I and J)** The knockdown efficiency of four different NBR1 ShRNA plasmids on the expression (I) of NBR1 in HEK293T cell lines and quantitative analysis (J). **(K-M)** Representative immunoblots showing the expression of IKKα, NF-κB p65, IκBα, p-IκBα and LC3 in HEK293T cell lines treated with NBR1 shRNA (K) and quantitative analysis (L and M). **(N)** IKKα associated with NBR1 and ANXA1 of primary microglial cells by immunoprecipitation with antibodies against IKKα. Data are expressed as mean ± SEM and analyzed by one-way ANOVA for (B, F, H, J, L and M) with Tukey's post hoc test or two-way ANOVA for (D) with Šídák's multiple comparison test. ns, not significant (p > 0.05), *p < 0.05, **p < 0.01, ***p < 0.001, and ****p < 0.0001.

Adult male wild-type C57BL/6 mice (8 weeks of age, 22-25 g) were provided by Vital River (Beijing, China) and used in Figure 1, Figure 2, Figure 3A-B, Figure S1 and Figure S3, aiming to explore the changes in different observation indicators under normal physiological conditions or MCAO surgery. Adult male Cx3cr1-Cre mice were obtained from The Jackson Laboratory (Stock#025524, USA), in C57BL/6 strain background and used in Figure 3H-I, Figure 4H-I, Figure 5A-B, Figure 7, Figure S5A-B, Figure S5H-J, Figure S6H, Figure S9 and Figure S10, aiming to verify the relationship between Tat-NTS peptide and ANXA1 SUMOylation in microglia, and the mice in the sham group and the MCAO surgery group that did not need virus injection, with the same batch as virus-injected mice, also used the same adult male Cx3cr1-Cre mice to ensure the same experimental state. To confirm knock-in of the Cre sequence, genomic DNA from tail was amplified using the primers 5'-CAACGAGTGATGAGGTTCGCAAG-3' and 5'-ACACCAGAGACGGAAATCCATCG-3'. Mice were bred in specific pathogen-free facility (~22 °C, 12:12 light/dark cycle) with access to water and food at all times. All experiments strictly followed the NIH Guide for the Care and Use of Laboratory Animals and approved by the Animal Care Committee of Tongji Medical College in Huazhong University of Science and Technology (Wuhan, China). Every effort was made to avoid animal suffering and to minimize the number of animals used throughout the experiments.

### Primary microglia culture and microglia isolation from adult mice

Mixed glial culture was isolated from the brain of 0- to 3-day-old neonatal mice, olfactory bulbs, cerebellum, midbrain and meninges were removed. The remaining brain tissue was minced and trypsinized to prepare a single cell suspension. Fetal bovine serum (FBS, Gibco) was added to stop the digestion. Cells were pelleted by centrifugation and resuspended in Dulbecco's modified Eagle's medium (DMEM, high-glucose, Gibco) containing 20% FBS (Gibco), then incubated in tissue culture flasks (Corning) precoated with poly-D-lysine (PDL, Sigma) for 8-10 days, and the medium was changed every 3 days. The microglia were separated through shaking the tissue culture flasks (200 rpm, 6 h, 37 °C) based on the differential adhesion of microglia and astrocytes and seeded into six-well plates (Corning) with a density of 1 × 10^6^ cells per well. The purity of primary microglia was greater than 95%, which was verified by immunostaining with specific microglia marker Iba1 (Figure S1B). For isolation of microglia from adult mice, microglial cells were purified by Percoll (GE Healthcare) density gradient centrifugation as previously described 12.

### Oxygen and glucose deprivation reperfusion (OGD/R) treatment

To model cerebral ischemia-reperfusion injury in vitro, cells were subjected to OGD/R treatment as described previously 12. Briefly, cells were rinsed three times with phosphate-buffered saline (PBS, Servicebio, Wuhan) and incubated in glucose-free DMEM (Gibco) without glucose and FBS at 37 °C. Then, cells were transferred to an anaerobic incubator with pre-mixed gas (95% N_2_ and 5% CO_2_, 37 °C) to establish OGD conditions. After 1 h of OGD, cells were transferred to normal DMEM with glucose and 10% FBS and placed in a normoxic incubator for 24 h to establish the OGD/R model.

### Protein extraction and preparation

Cell lysates were prepared from primary cultured microglia, neurons, HEK293T cells or brain tissue by RIPA lysis buffer (Beyotime, China) with protease inhibitor cocktail and phosphatase inhibitor cocktail (Roche). Nuclear and cytoplasmic extracts were prepared by the NE-PER™ Nuclear and Cytoplasmic Extraction Reagents (Thermo Scientific, #78833) according to the manufacturer's instructions. Protein concentrations were determined using an enhanced bicinchoninic acid (BCA) protein assay kit (Beyotime, China) prior to Western blot or immunoprecipitation analysis. Lysosomes were isolated by a lysosome isolation kit (Sigma) with density gradient ultracentrifugation according to the manufacturer's description. Brain tissue was prepared from the ipsilateral parietal cortex (penumbra of the middle cerebral artery) or hippocampus and the corresponding area of the contralateral side 20.

### LDH release, cell viability and cell survival assays

LDH released into the cell culture media as an indicator of lost membrane integrity and cell death. The culture medium from primary cultured microglia, was centrifuged and collected, and then assessed by LDH Cytotoxicity Assay Kit (Thermo Scientific, #88953) according to the manufacturer's instructions. The Cell Counting Kit-8 (CCK8, Beyotime, China) was used to evaluate cell viability for primary microglia. The absorbance at 450 nm was read using a microplate reader (Bio-Rad). To detect neuronal survival status, TUNEL staining was performed to assess cell survival using a cell death detection kit (Roche) as previously described 19. All above analyzes were performed in at least three independent experiments.

### Quantitative real-time PCR (qRT-PCR)

Total RNA was isolated with TRIzol reagent (Invitrogen), and complementary DNA (cDNAs) were synthesized by using ReverTra Ace-α-First Strand cDNA Synthesis Kit (Toyobo). qRT-PCR was performed with SYBR Green fluorescence on the StepOnePlus real-time PCR system (Applied Biosystems). The primers were designed using Primer 5 software (Table S2) and synthesized by Sangon Company (Shanghai, China). Gene expression levels were normalized to *β-actin* mRNA levels and determined by the 2^-ΔΔCt^ method.

### Enzyme-linked immunosorbent assay (ELISA)

ELISA kits (Dakewei, China) were applied to determine the cytokine concentrations of pro-inflammatory factors (IL-1β, IL-6 and TNF-α) and anti-inflammatory cytokines (IL-4, IL-10 and TGF-β) in the culture supernatant from primary cultured microglia with triplicate wells following the product instructions. As shown in Figure S2A and Figure S2C-D, the changes of the above microglia-derived cytokines under different conditions were detected by ELISA. The absorbance of ELISA plates was measured in a microplate reader (Bio-Rad) at 450 nm with deletion of background at 650 nm.

### Reagents and antibodies

The antibodies applied in this study were described in Table S1. CHX (Millipore, #508739) was purchased from Calbiochem (Germany). NH_4_Cl (Sigma, #A9434), 3-MA (Sigma, #M9281), CQ phosphate (Sigma, #PHR1258), MG-132 (Sigma, #C-2211), and Dimethyl sulfoxide (DMSO, Sigma, #D2650) were obtained from Sigma-Aldrich (Shanghai, China). All other reagents and solvents were commercially available, and were used as received unless otherwise noted.

### Immunoprecipitation and immunoblot analysis

After measuring the protein concentration, the extracted protein was incubated with antibodies overnight (4 °C) with IgG as a negative control. The mixtures were further captured on Protein A+G agarose beads (Beyotime, China) for 4 h at 4 °C, then washed four times with cold PBS and centrifuged (2500 rpm, 5 min) to clear any protein that may non-specifically adhered to the agarose beads. SDS sample buffer was then added and boiled for 10 min. Strips were resolved by SDS-PAGE and analyzed by immunoblotting. Gels were transferred to a PVDF membrane (Roche), then blocked with 5% bovine serum albumin (BSA), and incubated with the indicated antibodies overnight (4 °C). Finally, the appropriate horseradish peroxidase (HRP)-conjugated secondary antibodies (1:20000, Jackson) or light chain-specific secondary antibodies (#93702 or #58802, CST, 1:1000) and chemiluminescence detection kit (Pierce Thermo Scientific) were applied for immunodetection.

### Cell culture and transfection

HEK293T cells (ATCC, USA) were cultured in DMEM (10% FBS, 1% penicillin-streptomycin) at a humidified incubator (37 °C, 5% CO_2_). Transfections of the indicated plasmids carried out at 80-90% confluent cells using Lipofectamine 2000 (Invitrogen). All cells were tested for mycoplasma contamination and confirmed to be mycoplasma-free.

### Experiments with microglial-conditioned medium (MCM)

**Figure 6:**
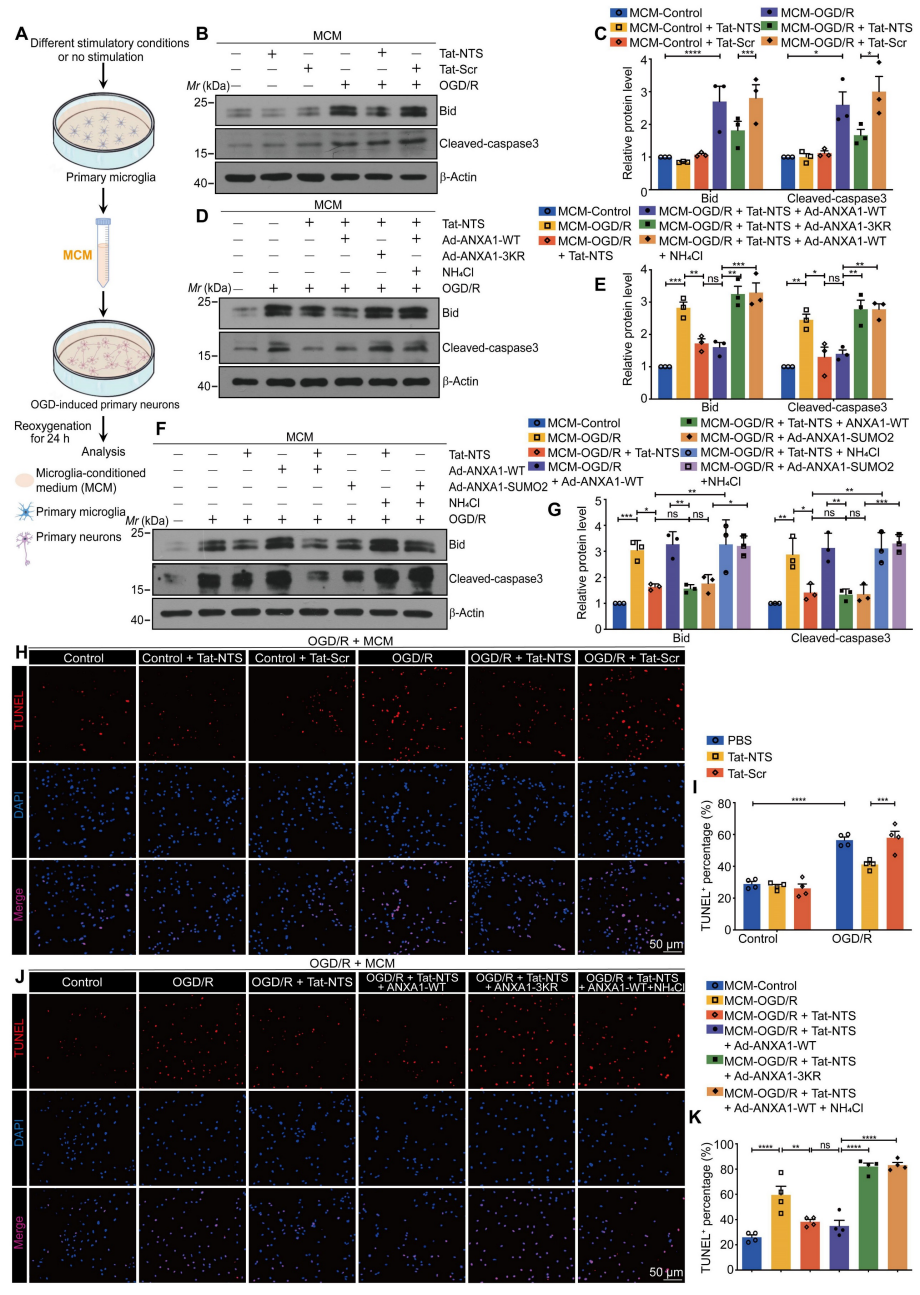
** Tat-NTS peptide attenuates OGD/R-induced neuronal apoptosis by regulating the effect of microglia-derived cytokines on neurons. (A)** Schematic diagram of the preparation of neuronal cells incubated with microglial conditioned media (MCM).** (B and C)** Immunoblots showing protein levels of Bid and cleaved-caspase3 in corresponding MCM-incubated primary cultured neurons (B) and quantitative analysis (C). **(D and E)** Representative blots for Bid and cleaved-caspase3 protein expression in corresponding MCM-incubated primary cultured neurons (D) and quantitative analysis (E).** (F and G)** Immunoblots showing protein levels of Bid and cleaved-caspase3 in corresponding MCM-incubated primary cultured neurons (F) and quantitative analysis (G).** (H and I)** Representative TUNEL staining images (H) and quantitative analysis (I) showing the effect of MCM from Tat-NTS peptide-treated microglia on neuronal apoptosis after OGD/R. Scale bar, 50 μm.** (J and K)** Representative TUNEL staining images (J) and quantitative analysis (K) showing the effect of MCM from ANXA1-3KR-overexpressed and Tat-NTS peptide-treated microglia on neuronal apoptosis upon OGD/R stimulation. Scale bar, 50 μm. Data are expressed as mean ± SEM and analyzed by one-way ANOVA for (C, E, G, and K) or two-way ANOVA for I with Tukey's post hoc test. ns, not significant (p > 0.05), *p < 0.05, **p < 0.01, ***p < 0.001, and ****p < 0.0001.

As shown in Figure 6A, Figure S2A, Figure S5G and Figure S7D, 6 h after OGD, the culture supernatant from primary microglia with different treatments was replaced with complete medium, and gently rinsed 3 times with sterile PBS at room temperature to exclude the effect of Tat-NTS peptide in the medium on ischemic neurons in subsequent experiments. The culture was continued until 24 h of reoxygenation after OGD, and then we collected this part of the medium supernatant as microglial-conditioned medium (MCM). The MCM from different treatment groups was centrifuged, and the supernatant was diluted 1:1 with fresh neuronal medium, and applied to neurons 21.

### Primary hippocampal neuronal cell culture

The hippocampus was dissociated from E18 mouse embryos, then minced and incubated in 0.25% trypsin-EDTA (37 °C, 10 min). DMEM with 10% FBS was then added to stop digestion. Then, the tissue was made into a single-cell suspension using a smooth glass pipette. Hippocampal neurons were centrifuged (1000 rpm, 5 min) and resuspended, then seeded into six-well plates (Corning) precoated with PDL at a density of 1 × 10^6^ cells per well. After 24 h of culture, the medium was replaced with Neurobasal (Gibco) medium containing 1% GlutaMAX (Gibco, #35050061), 2% B27 (Gibco, #17504044), and 1% penicillin-streptomycin (Gibco, #15070063), with half of the culture medium changed every 3 days until treatment. After 7-10 days, neurons were subjected to OGD for 1 h and the supernatant was replaced with MCM. The purity of primary hippocampal neurons was greater than 95%, as verified by immunostaining with the neuron-specific marker NeuN (Figure S8A).

### Microscopy analysis

Primary cultured microglia were seeded onto pre-coated glass coverslips, gently rinsed three times with pre-cooled PBS to remove residual medium, fixed in 4% paraformaldehyde (PFA) for 20 min, permeabilized with 0.3% Triton X-100 for 15 min, blocked with 5% donkey serum for 1 h, then incubated with specific primary antibodies (1:100 dilution) overnight at 4 °C. The next day, cells were incubated with secondary antibodies (1:200, Invitrogen) for 1 h at 37 °C, counterstained with DAPI for 10 min, and mounted for observation with a fluorescence microscope IX-73 (Olympus) and a Zeiss LSM780 laser scanning confocal microscope (Carl Zeiss). For staining of brain slices, 30-μm coronal frozen sections of mouse brain were prepared after fixation and dehydration, then permeabilized with 0.5% Triton X-100 for 30 min, followed by the same procedures as above for primary cultured microglia, and finally mounted for observation with a Zeiss LSM800 laser scanning confocal microscope (Carl Zeiss). Fluorescence images were analyzed and quantified using ImageJ Fiji software. To calculate the percentage distribution of nuclear and cytoplasmic fluorescence of ANXA1 or NF-κB p65, the fluorescence intensity of ANXA1 or NF-κB p65 inside and outside the nucleus was quantified by using the ImageJ polygon tool to select the corresponding region. All 3D reconstructions for z-stacks were generated from confocal microscopy and then transferred to Imaris software (Bitplane) to visually demonstrate protein localization and microglial morphology.

### RNA interference experiments

The shRNA sequences that target human NBR1 sequence (GenBank no. NM_001291571), the original shRNA targeting sequence for NBR1, 5′-GCCAGGAACCAAGTTTATCAA-3′ 22, was designed as follows: (1) 5'-TTAACTGCCATCTTAAGCGCTTCTT-3'; (2) 5′-TGATAAACTTGGTTCCTGGCT-3′; (3) 5′-TGATGATGTACACAAGGAGAGCTCC-3′; (4) 5′-ATTATTGGCTGTGCAGTGACTGGTG-3′. The vector expressing shRNAs was served as a negative control.

### Luciferase reporter assays

For reporter assays, HEK293T cells were seeded on 24-well culture plates (Corning) and transfected with the pGL3 plasmid containing the NF-κB luciferase reporter and the Renilla luciferase reporter plasmid (pRL-TK, Promega) co-transfected with plasmids expressing ANXA1 or the SUMO mutants of ANXA1. In addition, OGD/R stimulation, IKKα, IKKβ or IKKγ were used as different stimulus conditions. At 48 h after transfection, cells were lysed and luciferase and Renilla activities were measured according to the manufacturer's instructions for the dual luciferase assay (Promega). Firefly luciferase activity was normalized to Renilla luciferase activity.

### Plasmid construction

The Flag-tagged IKKα, IKKβ, IKKγ were generated by cloning the full-length cDNA into pCDNA3.1(+)-3×FLAG. The plasmid expressing the SUMOylation mutants of ANXA1 was constructed using the ClonExpress II One Step Cloning Kit (Vazyme, China) by homologous recombination. All plasmids were confirmed by Sanger sequencing (Sangon Biotech, China).

### Transient focal cerebral ischemia

The transient focal cerebral ischemia model was established by 1-h middle cerebral artery occlusion (MCAO) as follows. Briefly, male Cx3cr1-Cre mice (8 weeks old, 22-25 g) were anesthetized with a ketamine/xylazine (K/X) cocktail (100 mg/kg ketamine and 10 mg/kg xylazine, i.p.) 23, 24. A thermal blanket (Harvard Apparatus) was used to maintain body temperature at 37 °C during surgery. The left common carotid artery (CCA), internal carotid artery (ICA), and external carotid artery (ECA) were isolated after a midline incision (∼1 cm in length) in the neck; the CCA was carefully dissected and ligated, the ECA was isolated and ligated at two positions at the end of the ECA and near the ICA and ECA bifurcations, and then an arteriotomy was made to make a small incision, a silicone rubber-coated nylon monofilament with a diameter of 0. 22 mm was inserted approximately 10 mm into the ICA until resistance was encountered. Cerebral blood flow was monitored by laser Doppler flowmetry (PeriFlux 5000) to confirm successful occlusion; the monofilament remained in place for 1 h and was then gently withdrawn. As a control for the procedure, the sham-operated group underwent the same surgical procedure except for the filament embolization.

### Transmission electron microscopy (TEM)

Mice from different treatment groups were anesthetized and immediately perfused with precooled PBS, and the brains were immediately removed and fixed in 2.5% glutaraldehyde. The ipsilateral parietal cortex was then removed and fixed in 2.5% glutaraldehyde overnight (4 °C), postfixed in 1% OsO4 for 2 h, washed three times in cacodylate buffer, stained in 1% uranylacetate for 1 h, dehydrated in graded ethanol solutions, and embedded in Epon. Thin tissue sections were cut on an ultramicrotome (Leica) and placed on EM grids, poststained with uranium acetate and lead citrate, and examined under a Hitachi 7100 transmission electron microscope (Nikon). To quantify the number of autophagic vacuoles, 6 fields per mouse were calculated from 3 mice per treatment group. According to previous studies, autophagic vacuoles in tissue were identified and counted 20.

### Electrophysiology

Long-term potentiation (LTP) was performed as described previously 17. Briefly, mice were anesthetized and decapitated, then brains were rapidly removed and placed in precooled artificial cerebrospinal fluid (ACSF, pH 7.4 at 0-4 °C, oxygenated with 95% O_2_ and 5% CO_2_). Brain slices (300-μm thick) were rapidly cut on a vibratome (Leica) and incubated in oxygenated ACSF at 32 °C for 90 min, and then the field potential was recorded using a 64-channel planar multi-electrode recording setup (MED64, Alpha-Med Sciences). The fEPSPs recordings were made from CA1 neurons as the stimulation site in Schaffer collaterals and the input-output signal was recorded, and the stimulation intensity corresponding to 40% of the maximum response was chosen to evoke the fEPSPs. After the fEPSPs were stable (the range of variation in amplitude is less than 10% of the mean), a 4 × 1s 100-Hz stimulus was applied, and the fEPSPs were recorded for 90 min after the stimulus.

### Viral vector transduction

For adenovirus infection (multiplicity of infection 100), as in previous research 12. Primary cultured microglia were separately infected with corresponding Myc-tagged adenoviruses. For AAVs injections, Cx3cr1-Cre mice were randomly assigned to control or treatment groups, anesthetized with K/X cocktail and mounted onto a stereotaxic frame (RWD Life Science). A small hole was drilled through the skull and virus was injected into the cerebral cortex (from bregma; anteroposterior [AP]: 0.00 mm, lateral [L]: -2.05 mm, dorsoventral [DV]: -1.50 mm), striatum (from bregma; AP: 0.00 mm, L: -2.05 mm, DV: -3.50 mm) and CA1 region (from bregma; AP: -2.00 mm, L: -1.55 mm and DV: -1.55 mm) of hippocampus of the left hemisphere, at a rate of 50 nl/min (500 nl per injection site) with a 1 µl Hamilton microsyringe.

### 2, 3, 5-triphenyltetrazolium chloride (TTC) staining

The TTC staining was performed to determine the ischemic infarction 24 h after MCAO surgery. Briefly, male Cx3cr1-Cre mice (8 weeks old, 22-25 g) were euthanized after ischemia/reperfusion, and the brains were immediately collected, frozen and then cut into six 2-mm-thick slices, which were stained with 2% TTC solution (Sigma-Aldrich, 37 °C, 15-20 min) and then fixed in 4% PFA. Images were captured and the ischemic infarct area was measured on each coronal slice using ImageJ software (NIH). Finally, ischemic infarct size was calculated according to previous studies 12 and using the formula: infarct area × (contralateral hemisphere area/ipsilateral hemisphere area) to compensate for edema formation 25, 26.

### Modified neurological severity scores (mNSS) test

The mNSS test was used to evaluate the neurological functional outcome at 24 h after MCAO. The score was graded on a scale of 0 to 14 and included motor, sensory, reflex, and balance tests, with normal status scored as 0 and maximal deficit scored as 14. Cumulative scores of 10 to 14, 5 to 9, and 1 to 4 indicate severe, moderate, and mild injury, respectively. The mNSS test was performed by blinded independent examiners.

### Morris water maze (MWM) test

The MWM protocol was adapted from previously published reports 12. Briefly, the MWM test was performed in a circular tank (diameter, 1.2 m) containing opaque water (~22 °C) and a fixed platform (diameter, 6 cm), with different shapes positioned on the walls surrounding the tank used as spatial cues. Hidden platform training was performed with the platform hidden 1 cm underwater in the target quadrant for 7 consecutive days (4 trials starting in different quadrants per day). The escape latency of mice finding the platform was recorded. Tests were performed at the same time each day. Mice were allowed to search for the platform for 60 s, if they did not find the platform, they were led to the platform and kept there for 15 s. On day 9, a probe test was performed, the hidden platform was removed, and each mouse was allowed to swim for 60 s to find the platform. The latency to reach the platform, the swim paths, the time spent in the target quadrant during the probe test were recorded, and the number of times the mice crossed the platform area was counted.

### Open-field test (OFT)

The OFT was used to assess the locomotor activity of mice after surgery within an open field cubic box (50 × 50 × 30 cm^3^). The general locomotor activity of the animals was recorded for a 10-min testing period after habituation. The open field box was thoroughly cleaned with ethanol (70%) between two tested mice. To measure general activity variables, we measured the total distance explored and distance in the central area, time spent in the central area, mean speed, and number of entries into the central area.

### Novel object recognition task (NORT)

The NORT was used to measure hippocampus-dependent short-term memory retention and consisted of a training and a test session. Briefly, mice were allowed to explore the open field box for 30 min per day for habituation prior to testing. Mice were allowed to explore two identical objects (object 1 and 2) for 10 min during the training period. Mice were rested for 6 h, then the test session was conducted in which object 2 was replaced with a novel object of different surface texture, size, and shape. Mice were again housed and allowed to explore freely for 10 min. Mouse behavior was recorded using a digital automatic tracking system (Shanghai Xinruan, China).

### Rotarod test

The rotarod test was performed to assess motor coordination with a rotating cylinder (diameter, 6 cm) that was progressively accelerated. Mice were habituated to the test room for at least 30 min and given a 30-min training (5 to 10 rpm) before testing. Then, the time spent walking on top of the rod before falling off was recorded by the detector in testing session (4 to 40 rpm), with a maximum time of 5 min. Latency of each mouse to fall was repeated for four consecutive trials with an apart intra-trial interval of 30 min and averaged for subsequent analysis.

### Statistical analyses

Data were shown as the means ± standard error of the means (SEM) from at least three independent experiments. GraphPad Prism software (version 9.3.0.) was used for statistical analyses. Multiple group comparisons were analyzed by one-way or two-way analysis of variance (ANOVA) with appropriate post hoc tests. Discontinuous data were analyzed with the Kruskal-Wallis non-parametric test (Dunnett's post hoc test). Detailed statistical parameters for all data are described in Table S3, including definitions and exact values of n and statistical tests performed. A probability value (P-value) below 0.05 was considered statistically significant.

## Results

### Tat-NTS peptide shifts the subcellular localization of ANXA1 in microglia from nuclear to cytoplasmic localization after ischemic injury

Here, we found in 3D reconstructions that ANXA1 is mainly localized and uniformly distributed in the cytoplasm under physiological conditions, however, the expression of ANXA1 increased and translocated to the nucleus after mice underwent MCAO surgery, and the types of nerve cells with nuclear translocation of ANXA1 in the ischemic penumbra are mainly microglial cells at this time (Figure 1A and Figure S1A). This finding suggests that nuclear translocation of ANXA1 in microglia may play a deleterious role during the course of ischemic brain injury. Consistent with this, we found increased nuclear translocation of ANXA1 in primary cultured microglia upon OGD stimulation (Figure S1B-D). We hypothesized that blocking ANXA1 nuclear translocation might be a protective strategy for microglia in cerebral ischemic injury. Therefore, we applied Tat-NTS peptide, which is used to block ANXA1 nuclear translocation in neurons, and investigated whether Tat-NTS peptide is effective in microglia. We observed that increasing doses of Tat-NTS peptide significantly reduced ANXA1 nuclear translocation in primary microglia, and the threshold concentration for maximal effect was 20 μM (Figure S1E and F). To further determine the effective time window of pharmacological intervention, we treated primary cultured microglia with Tat-NTS peptide at a concentration of 20 μM immediately after OGD and terminated at five time points (2, 4, 6, 8 and 10 h after OGD treatment). Compared with the solvent control group, the initial effect of Tat-NTS peptide on ANXA1 nuclear translocation was observed at 2 h, with a maximum effect achieved at 6 h (Figure S1G-I). The time window and concentration that achieved the maximum effect (6 h, 20 μM) was used for subsequent in vitro experiments. Notably, Tat-NTS peptide had no cytotoxicity to normal microglia and did not affect cell viability under this condition, whereas it improved cell survival and reduced cytotoxicity after OGD/R (Figure S1J and K). This result suggests that Tat-NTS peptide has a good safety profile. Furthermore, we found that microglia transformed from a resting state with a ramified morphology to a typical amoeboid-like activated morphology 27-30 after MCAO/R surgery, accompanied by cytoplasmic ANXA1 aggregation towards the nucleus. After unilateral intracerebroventricular injection of Tat-NTS peptide (2 mg/kg) 19, the nuclear accumulation of ANXA1 was significantly reduced and relocated to the cytoplasm (Figure 1B-D and Figure S1L), the number of microglia branches increased and microglial morphology exhibited a beneficial transition from an activated to a resting state (Figure 1B and E) according to 3D reconstruction. These results demonstrate the efficient and excellent effect of Tat-NTS peptide in altering the subcellular localization of ANXA1 in microglia, and this shift in localization of ANXA1 in microglia may be closely related to the activation state of microglia.

### Tat-NTS peptide promotes a switch of microglia from a pro-inflammatory to an anti-inflammatory phenotype after ischemic stroke

To further investigate the biological significance of Tat-NTS peptide on microglial activation after ischemic injury, we examined the expression of several microglial phenotypic markers by qRT-PCR (Figure S2A). Encouragingly, we found that Tat-NTS peptide significantly abrogated the OGD/R-induced upregulation of pro-inflammatory associated genes *(IL-1β, IL-6, TNF-α, iNOS* and *CD16/32*) (Figure 2A and Figure S2B), and significantly increased the expression levels of anti-inflammatory associated genes (*Arg-1, TGF-β, IL-4, IL-10* and *CD206*) (Figure 2B and Figure S2B). Immunoblotting and ELISA were performed to measure the protein abundance and the protein secretion of pro-inflammatory and anti-inflammatory cytokines (Figure 2C-D and Figure S2C-D), and these results corresponded to the observed changes in mRNA expression levels. In addition, Tat-Scr peptide treatment had almost no effect compared to the untreated group, suggesting the specific effect of Tat-NTS peptide. Furthermore, in vivo experiments have also fully verified that Tat-NTS peptide treatment could significantly inhibit the expression of pro-inflammatory cytokines and upregulate the expression of anti-inflammatory cytokines in microglia after ischemic brain injury (Figure 2E-F and Figure S3A-E), triggering the microglial polarization to switch towards the repair anti-inflammatory phenotype.

### Tat-NTS peptide induces an increase in ANXA1 SUMOylation and suppresses ischemic injury-induced NF-κB activity of microglia through ANXA1 SUMOylation

Upon Tat-NTS peptide administration, we further quantified the expression of classic inflammatory markers IL-1β and TNF-α in microglia using immunoblotting (Figure S4A and B) and immunofluorescence (Figure S4C-E), which were initially increased under OGD conditions and then decreased after Tat-NTS peptide treatment. These results also corroborate that Tat-NTS peptide attenuates the microglial immune response. A similar effect has been demonstrated in previous studies of ANXA1 SUMOylation in microglial polarization and immune response 12. Meanwhile, ANXA1 SUMOylation occurred mainly in the cytoplasm (Figure S5A and B). These observations prompted us to further investigate whether Tat-NTS modulated ANXA1 SUMOylation. The result showed that the OGD/R-diminished interaction between HA-ANXA1 and His-SUMO2 in the cytoplasm was strongly reversed after treatment with Tat-NTS peptide (Figure S5C and D), and the interaction between endogenous ANXA1 and SUMO2 in OGD/R-treated microglia was found to be significantly upregulated under Tat-NTS peptide administration by an immunoprecipitation (IP) assay (Figure S5E and F). In addition, Tat-NTS peptide also upregulated ANXA1 SUMOylation in mouse microglia, which was reduced by ischemia (Figure 3A and B). Taken together, these data indicate that Tat-NTS peptide upregulates the SUMOylation levels of ANXA1 in the cytoplasm of ischemic injury-activated microglia.

To further investigate whether Tat-NTS peptide modulated microglial immune responses through ANXA1 SUMOylation, we used adenoviruses encoding Myc-tagged ANXA1-WT (wild-type ANXA1) and ANXA1-3KR (ANXA1 SUMOylation-deficient mutant) 12 to infect primary cultured microglia (Figure S5G). A dual-luciferase reporter assay was used to measure NF-κB p65 transcriptional activity, and the results indicated that Tat-NTS peptide profoundly inhibited OGD/R-mediated enhancement of p65 transcriptional activity, consistent with co-administration with ANXA1-WT, whereas co-administration with ANXA1-3KR abolished the positive effect (Figure 3C). Western blot analysis then revealed that Tat-NTS peptide strongly suppressed the phosphorylation and subsequent degradation of IκBα induced by OGD/R stimulation. In contrast, simultaneous impairment of ANXA1 SUMOylation (ANXA1-3KR) dramatically reversed this effect (Figure 3D and E).

Consistent with this, we further manipulated gene overexpression in microglia/macrophages from specific regions of the adult male Cx3cr1-Cre mice (Figure S5H), both in cultured microglia and in vivo microglia, Tat-NTS peptide treatment caused a marked downregulation in the expression of the pro-inflammatory NF-κB targets IL-1β and TNF-α in microglia subjected to ischemic injury, whereas co-treatment with ANXA1-3KR overexpression showed an opposite effect (Figure 3F-I and Figure S5I-J). The above experiments demonstrated that the protective effect of treatment with Tat-NTS peptide alone, was similar to that of ANXA1-WT overexpression on this basis, whereas co-administration with ANXA1-3KR overexpression showed the opposite or even more severe effect. This suggests that Tat-NTS peptide suppresses ischemic injury-induced microglial immune response and NF-κB activity, whereas the effect is antagonized when SUMOylation of ANXA1 is blocked.

### Tat-NTS peptide promotes IKKα protein degradation via SUMOylated ANXA1 to inhibit cerebral ischemic injury-mediated NF-κB p65 nuclear translocation

To further define the precise molecular mechanism by which Tat-NTS peptide restricts ischemic injury-mediated NF-κB activation, we extended the search for the upstream signaling proteins of the NF-κB pathway. Plasmids expressing Flag-tagged IKKα, IKKβ or IKKγ were transfected separately into HEK293T cells, while an NF-κB luciferase reporter vector was added along with Tat-NTS peptide treatment. The results indicated that IKKα overexpression partially antagonized the inhibitory effect of Tat-NTS peptide on NF-κB transcriptional activation, whereas IKKβ or IKKγ overexpression had no apparent effect (Figure S6A). This difference suggested that the IKK subunit might be a promising upstream target. Therefore, we used a co-immunoprecipitation (co-IP) assay to examine the interaction of different subunits with ANXA1 under Tat-NTS peptide treatment, and the results revealed that ANXA1 bound only IKKα under Tat-NTS peptide treatment (Figure S6B). Interestingly, we found that Tat-NTS peptide significantly reduced the OGD/R-induced upregulation of IKKα protein levels, but had no effect on IKKβ or IKKγ (Figure 4A and B). Furthermore, we observed that Tat-NTS peptide could promote the binding between endogenous IKKα and ANXA1 in microglia by co-IP analysis, as expected. In contrast, the interaction of endogenous ANXA1 with IKKα was strongly inhibited after ANXA1-3KR treatment (Figure 4C). Taken together, these results confirm that Tat-NTS peptide targets IKKα to regulate OGD/R-induced NF-κB activation. Further investigation of the mRNA level of the IKKα encoding gene* CHUK* in primary microglia was performed by qPCR and showed that Tat-NTS peptide had no effect on *CHUK* mRNA expression (Figure S6C). The decrease of IKKα abundance occurred at the proteomic rather than the transcriptomic level. Since protein degradation is one of the major mechanisms regulating protein levels 31, we assessed the degradation of IKKα using a cycloheximide (CHX) chase assay. As expected, Tat-NTS peptide shortened the protein half-life of endogenous IKKα in OGD/R-stimulated microglia, but this effect was reversed by ANXA1-3KR treatment (Figure 4D and E). Finally, we determined the effect of Tat-NTS peptide on NF-κB p65 nuclear translocation associated with IKK complex. Immunoblot analysis of cytoplasmic and nuclear fractions of NF-κB p65 in microglia indicated that Tat-NTS peptide robustly alleviated OGD/R-induced nuclear accumulation of NF-κB p65, but ANXA1-3KR treatment strongly antagonized the effects of Tat-NTS peptide (Figure 4F-G and Figure S6D). Consistently, immunofluorescence assay showed that ischemic injury-mediated nuclear translocation of NF-κB p65 in both cultured and in vivo microglia was blocked by Tat-NTS peptide and then enhanced by ANXA1-3KR (Figure 4H-I and Figure S6E-H). Taken together, these results demonstrate that Tat-NTS peptide promotes IKKα degradation in microglia to downregulate the activation of downstream NF-κB pathways under cerebral ischemic injury. This process is dependent on ANXA1 SUMOylation.

### Tat-NTS peptide facilitates NBR1-dependent selective autophagic degradation of IKKα

Similarly, we observed in vivo that Tat-NTS peptide promoted the degradation of endogenous IKKα after ischemic injury (Figure 5A and B). Next, we used pharmacological approaches to confirm the clearance system for IKKα degradation, including the proteasome inhibitor N-carbobenzyloxy-l-leucyl-l-leucyl-l-leucinal (MG-132), the autophagy inhibitors such as chloroquine (CQ), 3-methyladenine (3-MA) and the lysosomal inhibitor ammonium chloride (NH_4_Cl). As shown in Figure S7A and S7B, Tat-NTS peptide-mediated degradation of IKKα was entirely blocked by the autophagy inhibitors, but not by the proteasome inhibitor. The autophagy inhibitors also suppressed Tat-NTS-mediated endogenous IKKα clearance in microglia after OGD/R (Figure 5C and D), suggesting that Tat-NTS peptide enhances IKKα degradation through the autophagy-lysosome pathway. Light chain 3-II (LC3-II) is the most reliable autophagy marker so far, and the ratio of LC3-II/LC3-I is commonly used to assess autophagy 32, 33. As expected, the ratio of LC3-II/LC3-I was dramatically increased by Tat-NTS peptide, notably, co-administration of ANXA1-3KR or the lysosomal inhibitor NH_4_Cl inhibited the increased ratio (Figure 5E and F), suggesting that Tat-NTS peptide induced autophagic flux. In addition, the accumulation of IKKα in lysosomes was detected by immunoblotting and showed that IKKα was present in isolated lysosomes to a certain extent, Tat-NTS peptide treatment gradually increased the abundance of IKKα in lysosomes (Figure 5G and H). Collectively, these results further support the contention that Tat-NTS peptide-mediated IKKα degradation occurs through the autophagy-lysosome pathway and that IKKα can be degraded by selective autophagy. Selective autophagy receptors, such as NBR1, normally recognize ubiquitinated cargo and deliver specific cargo to lysosomes/vacuoles for degradation of ubiquitinated substrates in mammals 34, 35. Next, we constructed a specific short hairpin RNA (shRNA) to knock down endogenous NBR1 (Figure 5I-J and S7C), and the immunoblotting revealed that Tat-NTS peptide-mediated IKKα-selective autophagic degradation was diminished by NBR1 shRNA. Notably, treatment with NBR1 shRNA strongly blocked Tat-NTS peptide-mediated degradation of IKKα and downregulated autophagy levels, with similar negative effects as treatment with ANXA1-3KR (Figure 5K-M). These results suggest that NBR1 is a necessary element for Tat-NTS peptide-regulated selective autophagic degradation of IKKα.

To investigate the interactions between NBR1, IKKα and ANXA1 in microglia, we performed IP experiments and found that the interaction between these proteins was decreased after OGD/R together with ANXA1-WT treatment, and this interaction was significantly enhanced by Tat-NTS peptide or ANXA1-SUMO2 treatment alone (Figure 5N and Figure S7D). Finally, we further verified the upstream/downstream relationships involved in the mechanism of action of Tat-NTS peptide in primary cultured microglia by immunoblotting. OGD/R-induced upregulation of IKKα and subsequent activation of the NF-κB pathway was aggravated by simultaneous overexpression of ANXA1; on this basis, the addition of Tat-NTS peptide significantly reversed the OGD-mediated injury. Tat-NTS peptide or ANXA1-SUMO2 treatment showed similar ischemia-protective effects, both involving upregulation of autophagy levels and downregulation of NBR1 expression. However, this protective effect and autophagy activation were significantly blocked after NH_4_Cl treatment (Figure S7E-G). In conclusion, Tat-NTS peptide enhances ANXA1 SUMOylation-mediated IKKα degradation through NBR1-dependent selective autophagy in microglia, thereby inhibiting NF-κB pathway activation and reducing pro-inflammatory cytokine release.

### Tat-NTS peptide exerts neuroprotective effects by modulating the effect of microglia-derived cytokines on neuronal apoptosis

To further elucidate whether Tat-NTS peptide can protect neurons from ischemic damage by alleviating microglia activation, as shown in Figure 6A, glucose-free medium was replaced with microglial-conditioned medium (MCM) immediately after OGD of neurons 21, 36. We observed a beneficial change in microglia-derived pro-inflammatory cytokines under Tat-NTS peptide treatment (Figure S2A, C and D), and based on this, the expression of the pro-apoptotic Bid and the apoptosis executioner cleaved-caspase3 37, 38 were detected to determine the apoptosis levels of ischemic neurons by immunoblotting.

Compared with incubation with MCM collected from the control Tat-Scr peptide-treated microglia, MCM collected from microglia which were treated with OGD/R and Tat-NTS peptide, significantly inhibited OGD/R-induced neuronal apoptosis, as expected (Figure 6B-C and Figure S8A). More importantly, the neuroprotective effect of Tat-NTS peptide treatment in microglia was completely antagonized by ANXA1-3KR overexpression (Figure 6D and E). MCM collected from AXNA1-SUMO2 overexpression microglia had almost the same protective effect as that derived from Tat-NTS peptide-treated microglia, and both could be reversed by the addition of NH_4_Cl on this basis (Figure 6F and G). Consistent with the changes in the expression of pro-apoptotic proteins, the number of TUNEL-positive apoptotic neurons was significantly increased after incubation with the MCM derived from OGD/R-treated microglia, and the percentage of apoptotic neurons was decreased when the MCM was derived from Tat-NTS peptide or ANXA1-SUMO2-treated microglia (Figure 6H-I and Figure S8B-C), whereas treatment of microglia with ANXA1-3KR or NH_4_Cl on this basis completely eliminated or even worsened this neuroprotective effect (Figure 6J-K and Figure S8B-C). The findings in primary neurons further validate the hypothesis that microglial activation after ischemic injury affects the status of neighboring neurons by releasing pro-inflammatory cytokines into the extracellular milieu, whereas Tat-NTS peptide can preserve neuronal survival after OGD/R by reducing the release of pro-inflammatory cytokines in microglia.

### Tat-NTS peptide improves neurological function after focal ischemic injury via ANXA1 SUMOylation in microglia

**Figure 7:**
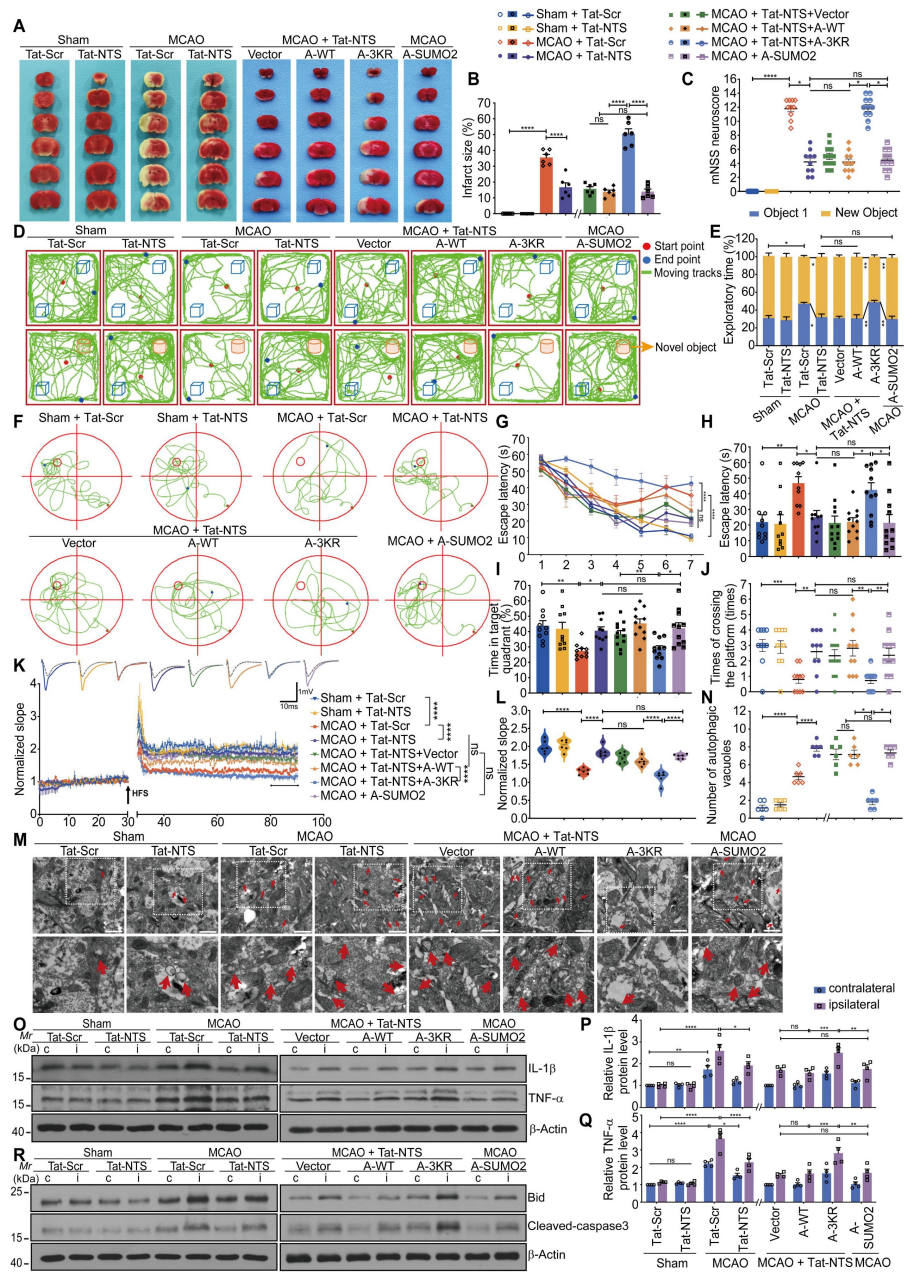
** Tat-NTS peptide rescues ischemia-mediated brain injury and improves neurobehavioral function by microglial ANXA1 SUMOylation-dependent after focal ischemic injury. (A and B)** Cerebral ischemic infarct size was assessed by TTC staining at 24 h after reperfusion (A) and quantitative analysis of infarct size (B). To facilitate image layout, we use abbreviations: A-WT, ANXA1-WT; A-3KR, ANXA1-3KR; A-SUMO2, ANXA1-SUMO2. **(C)** Neurological outcome was assessed by mNSS at 24 h after reperfusion. **(D)** Representative traces from each group in the novel object recognition task (NORT).** (E)** Percentage of time spent exploring the familiar and novel objects in the test session. **(F)** Representative swimming traces from different groups of mice during probe trials on day 9.** (G)** Latency to reach the hidden platform during training trials. **(H)** Latency to first arrival at the hidden platform on day 9. **(I)** Percentage of time spent in the target quadrant on day 9. **(J)** Number of times mice crossed the platform area on day 9. **(K and L)** Long-term potentiation (LTP) of the hippocampus was inhibited after MCAO/R but rescued by Tat-NTS peptide, the average normalized slope (K) and quantitative analysis of the fEPSPs of the last 10 min indicated in (K) are shown (L). HFS, high-frequency stimulation; gray dotted line, before HFS induction; colored solid line, after HFS induction. n = 6 slices per group. **(M)** Representative TEM images of the ultrastructure of autophagic vacuoles (red arrow) in the cytoplasm of cortical microglia are shown. Scale bar, 1 μm. **(N)** The quantified number of autophagic vacuoles per TEM field from six randomly selected fields. **(O-Q)** Representative immunoblots showing the protein levels of proinflammatory cytokines IL-1β and TNF-α in the cortical penumbra and contralateral cortical tissue of the adult male Cx3cr1-Cre mice (O) and quantitative analysis (P and Q). **(R)** Protein expression of apoptotic factors Bid and cleaved-caspase3 in the bilateral hippocampus of the Cx3cr1-Cre mice was measured by immunoblot analysis. N = 10-12 mice per group in (C-J); n = 3-6 mice per group in (A, B and K-R). Data are expressed as mean ± SEM and analyzed by one-way ANOVA for (B, C, H, I, J, L and N) or two-way ANOVA for (E, G, K, P and Q) with Tukey's post hoc test or Dunnett's post hoc test (C, J and N). *p < 0.05, **p < 0.01, ***p < 0.001, and ****p < 0.0001, ns, not significant (p > 0.05).

To further verify whether Tat-NTS peptide affects neurofunctional outcome in ischemic mice through ANXA1 SUMOylation in microglia, we injected adeno-associated viruses (AAVs) carrying ANXA1-WT, ANXA1-3KR or ANXA1-SUMO2 into the cerebral cortex, striatum and hippocampal CA1 region of adult male Cx3cr1-Cre mice, and performed histological, behavioral and electrophysiological experiments at the designated time points after reperfusion (Figure S9A-E). After unilateral intracerebroventricular injection of Tat-NTS peptide (2 mg/kg), ischemic infarct size was quantified by TTC staining (Figure 7A and B), and neurological function was evaluated by the modified Neurological Severity Score (mNSS) test (Figure 7C) after surgery, the Tat-NTS peptide-treated group showed a significantly reduced ischemic area and neurological deficit scores compared with the control peptide-treated group. However, administration of Tat-NTS peptide under the premise of ANXA1-3KR overexpression reversed the Tat-NTS peptide-mediated improvement in nerve damage. To further observe the effect of Tat-NTS peptide treatment on spontaneous locomotor activity and motor coordination in ischemic mice, we performed the open field test (OFT) (Figure S10A-G) and the rotarod test (Figure S10J). Similarly, Tat-NTS peptide-treated mice showed a dramatic improvement in motor function and significantly more spontaneous locomotor activity, consistent with ANXA1-WT pre-injection or ANXA1-SUMO2 injection alone (Figure S10A-G and J). In addition, the amelioration of cognitive function in Tat-NTS peptide-treated mice after focal ischemic injury was assessed by the novel object recognition task (NORT). In the test trial, ANXA1-3KR abolished the better novel object preference exhibited by Tat-NTS peptide (Figure 7D-E and Figure S10H). Afterwards, we performed the Morris water maze (MWM) test to assess the spatial learning and memory abilities of the mice after MCAO surgery. Tat-NTS peptide-treated mice showed significant cognitive improvement (Figure 7F-J and Figure S10I), including latency to the first platform crossing (Figure 7G and H), time spent in the target quadrant (Figure 7I), and total number of platform crossings (Figure 7J). Consistently, synaptic plasticity was assessed by analyzing long-term potentiation (LTP) in the hippocampus (Figure 7K and L), and the results showed that synaptic plasticity was significantly enhanced in Tat-NTS peptide-treated mice compared to the Tat-Scr peptide-treated group after ischemia-reperfusion. As expected, administration of Tat-NTS peptide alone, or administration of Tat-NTS peptide under the premise of overexpression of ANXA1-WT, or only overexpression of ANXA1-SUMO2 in microglia of model mice, can greatly protect against ischemia-induced neurological impairment. The neuroprotection induced by Tat-NTS peptide was completely blocked or even worsened by overexpression of ANXA1-3KR in microglia.

Finally, the number of autophagic vacuoles (Figure 7M and N), the protein expressions of inflammatory factors (Figure 7O-Q) and apoptotic factors (Figure 7R and Figure S10K-L) in the ischemic side were sequentially detected under different treatment conditions. Transmission electron microscopy (TEM) was used to observe the number of autophagic vesicles and to evaluate the ultrastructure of microglia from the cerebral cortex. As shown in Figure 7M and Figure 7N, Tat-NTS peptide treatment increased autophagy levels in cortical microglia after focal ischemic injury in mice. The protein expressions of ischemia-induced pro-inflammatory factors IL-1β and TNF-α (Figure 7O-Q) and apoptosis indicator Bid and cleaved-caspase3 (Figure 7R and Figure S10K-L) were considerably diminished in the ischemic side of Tat-NTS peptide-treated mice. These data showed that administration of Tat-NTS peptide, treatment with Tat-NTS peptide under the premise of overexpression of Vector or ANXA1-WT, both similar to that of overexpression of ANXA1-SUMO2 alone, had analogous anti-ischemic, autophagy-promoting, anti-apoptotic, anti-inflammatory properties in the model mice. In contrast, this neuroprotective effect was reversed and further damage was observed when ANXA1 was unable to undergo SUMOylation (ANXA1-3KR), and administration of Tat-NTS peptide under the premise of ANXA1-3KR overexpression exerted a clear negative effect of aggravating ischemic injury. Taken together, these in vivo data demonstrate that Tat-NTS peptide improves tissue and functional outcome after ischemic brain injury, rescues impaired cognitive and motor function, and thus exerts powerful neuroprotective effects in ischemic injury mice by modulating ANXA1 SUMOylation in microglia/macrophages.

## Discussion

**Figure 8:**
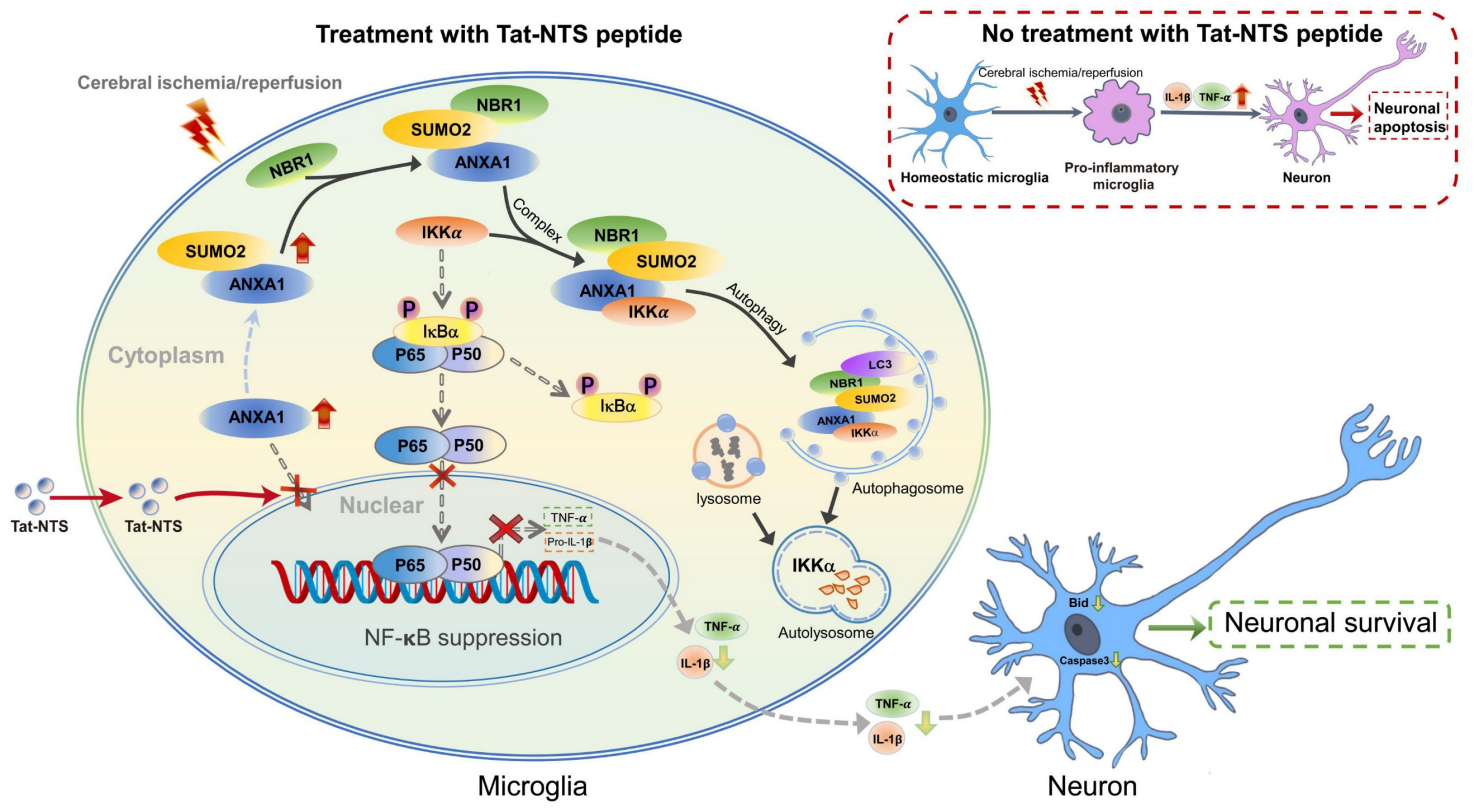
** Schematic depicting strategies for Tat-NTS peptide to protect against ischemic stroke-induced neuronal cell apoptosis via inhibition of microglial inflammatory response.** The schematic diagram summarizes the main findings of the present study. Tat-NTS peptide blocked the nuclear translocation of ANXA1 in microglia stimulated by cerebral ischemic injury. ANXA1 remained in the cytoplasm and was modified by SUMOylation and then associated with IKKα and NBR1, leading to autophagic degradation of IKKα, further suppressing the NF-κB pathway and reducing the release of microglia-derived proinflammatory mediators IL-1β and TNF-α, thereby attenuating neuronal apoptosis and facilitating the recovery of neurological function after cerebral ischemic injury.

This study provides a novel mechanistic insight into how Tat-NTS targets microglial ANXA1 to protect against ischemic brain injury (Figure 8). We used Tat-NTS peptide to effectively block the nuclear translocation of ANXA1 in microglia, and further observed the effect of Tat-NTS peptide on microglia by promoting the cytoplasmic localization of ANXA1 after focal cerebral ischemia. Interestingly, we observed that Tat-NTS peptide modulated ischemic brain injury-induced microglial polarization towards the anti-inflammatory phenotype and robustly decreased the expression of pro-inflammatory mediators IL-1β and TNF-α in ischemia-stimulated microglia. Our previous research has demonstrated that ANXA1 in the cytoplasm of microglia can be SUMOylated by SUMO2, and the SUMOylated ANXA1 exhibited cytoplasmic localization and promoted microglial transformation towards the anti-inflammatory phenotype 26. These findings are consistent with the fact that Tat-NTS peptide enables ANXA1 to accumulate in the cytoplasm and attenuates the inflammatory response of primary microglia subjected to MCAO/R. The IP results indicated that the OGD/R-induced decrease in cytoplasmic ANXA1 SUMOylation was significantly upregulated by Tat-NTS. We then used adenoviruses encoding ANXA1-3KR, which has been shown to effectively and completely block ANXA1 SUMOylation, to infect primary cultured microglia, and detected IκBα phosphorylation and degradation to assess NF-κB pathway activation. The results showed that Tat-NTS peptide suppressed NF-κB pathway activation, whereas co-administration with ANXA1-3KR reversed the protective effect or even caused more severe damage. This suggests that Tat-NTS peptide inhibits OGD/R-induced NF-κB activity via ANXA1 SUMOylation. The IKK complex is required for IκBα phosphorylation and degradation, consisting of catalytic (IKKα and IKKβ) and regulatory (IKKγ/NEMO) subunits 39, 40. Specifically, we observed that Tat-NTS peptide promoted IKKa degradation and then blocked NF-κB p65 nuclear translocation in vivo, and further clarified that Tat-NTS peptide facilitated NBR1-dependent selective autophagic degradation of IKKα by upregulating ANXA1 SUMOylation. In particular, our results revealed that Tat-NTS peptide treatment attenuated ischemia/reperfusion injury-induced neuronal apoptosis through inhibiting the release of microglia-derived pro-inflammatory cytokines. Finally, histological, behavioral and electrophysiological results indicated that Tat-NTS peptide modulated ANXA1 SUMOylation in microglia/macrophages and dramatically improved neurofunctional outcome in animals with ischemic brain injury.

The Tat (YGRKKRRQRRR) protein is a member of the human immunodeficiency virus (HIV)-derived cationic cell-penetrating peptides (CPPs), which can transport proteins across the plasma membrane into cells 41. Tat-mediated transduction has emerged as a promising therapeutic approach for cerebral ischemic stroke and other CNS disorders 42, 43. For example, Tat-NR2B9c has been shown to be safe and effective in ameliorating ischemic brain injury in cynomolgus monkeys and humans 19. Tat-CIRP is able to inhibit TLR4 signaling by interfering with myeloid differentiation protein-2 (MD2) and reduce infarct volume in non-human primates without evidence of toxicity 44. It is worth noting that Tat-NTS peptide has been experimentally proven to exert neuroprotective effects in both OGD/R models and MCAO models of stroke, and that Tat-NTS peptide-mediated effects were injury specific, with no apparent effect on neuronal apoptosis or cognitive function in non-ischemic (sham-operated) animals. Taken together, these findings demonstrate that Tat-NTS peptide has high efficacy, safety and potential for clinical translation and application.

SUMOylation, which is one of the most important post-translational modifications of proteins, is involved in various biological processes such as transcriptional regulation, signal transduction and maintenance of protein stability 12. One of the candidate pathways against ischemic injury is the post-translational modification of target proteins with SUMO. Interestingly, recent works suggest a critical role for SUMOylation in the human penumbra 45, 46. The major SUMO modification sites of ANXA1 (K113/161/257) are highly conserved among its homologs in different species 12. Our study demonstrated that Tat-NTS peptide upregulated ANXA1 SUMOylation and promoted microglial transformation towards the anti-inflammatory phenotype to rescue ischemia-induced neuronal apoptosis. This observation inspired us to consider the broader application prospects for Tat-NTS peptide.

The IKK/NF-κB signaling pathway is a key regulatory mechanism of inflammatory responses 47-49, and the protective role of autophagic degradation of the IKK complex has been validated in many biological processes. For example, the ANXA1 peptide Ac2-26 promotes IKKβ degradation within lysosomes by chaperone-mediated autophagy, thereby reducing TNF-α expression 50. NBR1-mediated autophagy is relatively common in animals and plants and usually plays a protective role 51-53. Consistently, this study found that Tat-NTS peptide promoted NBR1-dependent selective autophagic degradation of IKKα, thereby inhibiting ischemic brain injury-mediated NF-κB activity and the release of pro-inflammatory cytokines IL-1β and TNF-α.

## Conclusions

Our study reveals a previously unrecognized molecular mechanism of Tat-NTS peptide function, whereby Tat-NTS peptide administration suppresses the release of pro-inflammatory factors from microglia and alleviates neuronal apoptosis, thereby protecting against cerebral ischemia-reperfusion injury. Our previous work has confirmed that Tat-NTS peptide has long-lasting therapeutic efficacy with a considerable therapeutic window (> 6 h) after focal ischemic stroke 19. In addition, this study further demonstrates the high efficacy (effective in 2 h) and safety of Tat-NTS peptide application. Further work to assess the neuroprotective effects and therapeutic targeting of Tat-NTS peptide administered by other peripheral routes, such as oral, subcutaneous, intraperitoneal and intravenous injections, is also of great clinical importance and ongoing studies in our laboratory are addressing these issues.

## Supplementary Material

Supplementary figures and tables.

## Abbreviations

AAVs: adeno-associated viruses
ANOVA: analysis of variance
ANXA1: Annexin-A1
ANXA1-WT: wild-type ANXA1
Arg-1: arginase-1
CD16/32: Fc gamma RII/RIII
CHX: cycloheximide
Co-IP: Co-immunoprecipitation
CPPs: cationic cell-penetrating peptides
CQ: chloroquine
DMEM: Dulbecco's modified Eagle's medium
ELISA: Enzyme-linked immunosorbent assay
HIV: human immunodeficiency virus
HRP: appropriate horseradish peroxidase
IL-1β: interleukin-1β
IL-4: interleukin-4
IL-6: interleukin-6
IL-10: interleukin-10
iNOS: inducible nitric oxide synthase
LC3-II: light chain 3-II
LDH: lactate dehydrogenase
LTP: long-term potentiation
MCAO: middle cerebral artery occlusion
MCM: microglial-conditioned medium
MG-132: N-carbobenzyloxy-l-leucyl-l-leucyl-l-leucinal
mNSS: modified neurological severity score
MWM: Morris water maze
NORT: novel object recognition task
NTS: nuclear translocation signal
OFT: open field test
OGD/R: oxygen-glucose deprivation and reperfusion
P-value: probability value
qRT-PCR: quantitative real-time PCR
SEM: standard error of the means
shRNA: short hairpin RNA
SUMO: small ubiquitin-related modifier
Tat: transactivator of transcription
TEM: transmission electron microscopy
TGF-β: transforming growth factor-β
TNF-α: tumor necrosis factor-α
tPA: tissue-plasminogen activator
TTC: 2, 3, 5-triphenyltetrazolium chloride
TUNEL: TdT-mediated dUTP nick-end labeling
