# Supplementary material for: Tat-NTS peptide protects neurons against cerebral ischemia-reperfusion injury via ANXA1 SUMOylation in microglia
Source: Theranostics. 2023 Oct 16;13(15):5561–83. doi: 10.7150/thno.85390 (PMC10614677; doi:10.7150/thno.85390)
Supplement: Supplementary file 1 — Supplementary figures and tables. [file thnov13p5561s1.pdf]

Supplementary Materials for

Tat-NTS peptide protects neurons against cerebral ischemia-reperfusion injury via ANXA1 SUMOylation in microglia

Huijuan Zhou[1,2,3], Lulu Yan[1,2,3], Hezhou Huang[2,3,6], Xing Li[4], Qian Xia[4], Lu Zheng[5], Bin Shao[1,2,3], Qian Gao[1,2,3], Ning Sun[1,2,3,*], Jing Shi[1,2,3,*]

*Correspondence author. E-mail: Jing Shi sj@mails.tjmu.edu.cn; Ning Sun nsun@hust.edu.cn

This PDF file includes:

Figure S1. Tat-NTS peptide reduces ANXA1 nuclear translocation in microglia after ischemic injury.

Figure S2. Tat-NTS peptide inhibits the expression of pro-inflammatory cytokines and upregulates the expression of anti-inflammatory cytokines in microglia after OGD/R.

Figure S3. Tat-NTS peptide induces microglial polarization after ischemic injury to switch towards the reparative anti-inflammatory phenotype.

Figure S4. Tat-NTS peptide decreased the expression of classic inflammatory markers IL-1β and TNF-α in OGD/R treated microglia.

Figure S5. Tat-NTS peptide upregulates the SUMOylation level of ANXA1 in the cytoplasm after ischemic injury and suppresses the expression of ischemic injury-induced pro-inflammatory factors IL-1β and TNF-α by ANXA1 SUMOylation.

Figure S6. Tat-NTS peptide enhances IKKα degradation and blocks nuclear translocation of NF-κB p65 after ischemic injury.

Figure S7. Tat-NTS peptide enhances degradation of IKK α through NBR1-dependent selective autophagy.

Figure S8. Microglia treated with Tat-NTS peptide improves neuronal survival under OGD/R conditions.

Figure S9. AAVs transfection manipulate gene overexpression in microglia/macrophages from specific regions of mice.

Figure S10. Tat-NTS peptide-treated mice show improved motor and cognitive function, and inhibited apoptosis levels after ischemic cerebral injury.

Table S1. Antibodies used in this study.

Table S2. Primers used in this study.

Table S3. Statistical analyses for all figures.

Supplementary Figures

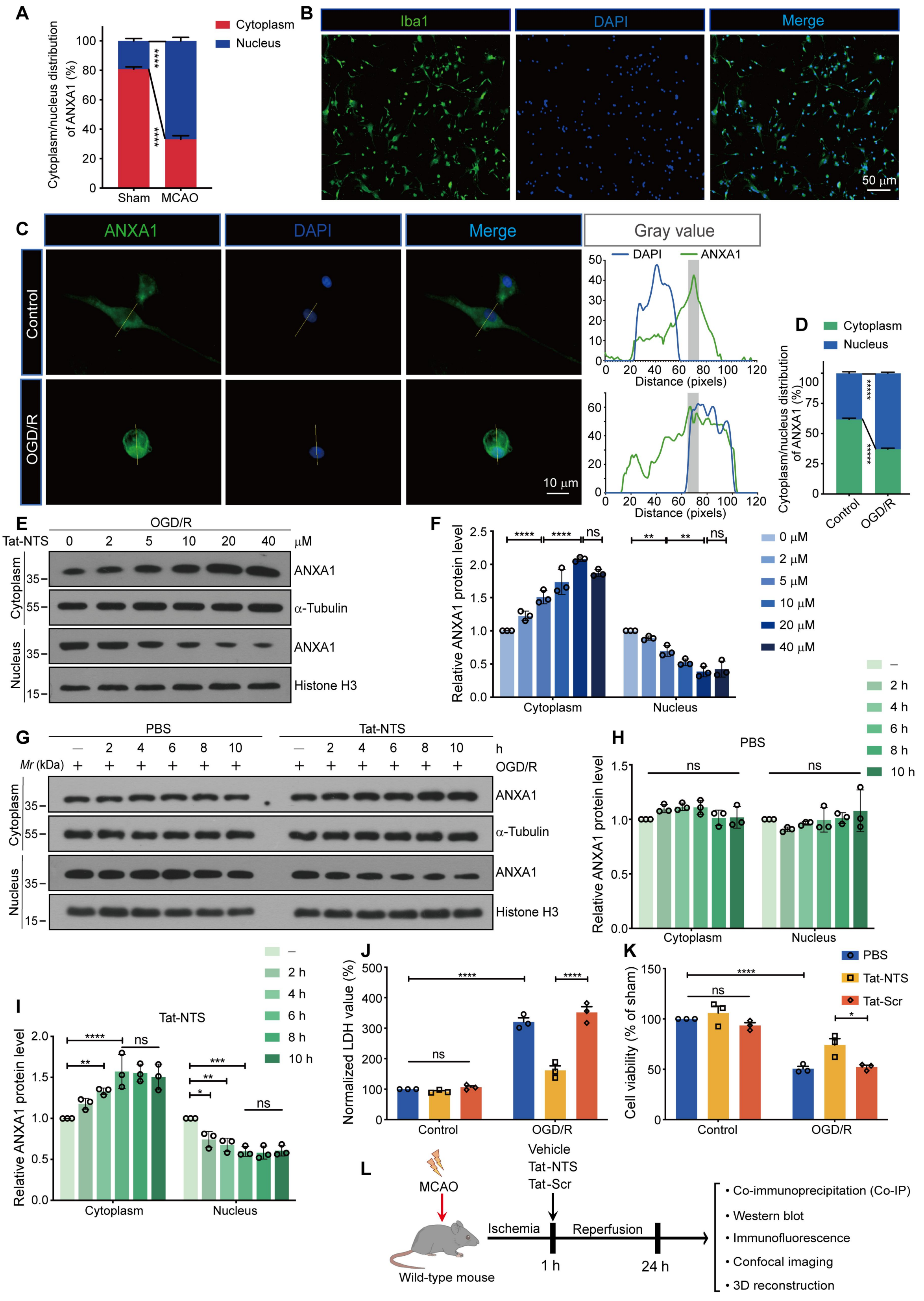

Figure S1. Tat-NTS peptide reduces ANXA1 nuclear translocation in microglia after ischemic injury.

(A) Quantitative analysis of the percentage distribution of nucleocytoplasmic fluorescence of ANXA1 in Figure 1A using Fiji ImageJ software. **(B)** Immunofluorescence analysis shows the purity of primary cultured microglial cells. Cells were stained with the microglia-specific marker Iba1 (green). Scale bar, 50 μm. **(C)** Fluorescence co-localization of ANXA1 (green) and DAPI (blue) in primary microglia after OGD/R. Merged profiles of fluorescence intensity of ANXA1 (green line) and DAPI (blue line) signals along the lines crossing the cells as indicated on the right in (C). Light gray areas indicate the ANXA1 peak. Scale bar, 10 μm. **(D)** Quantitative analysis of the percentage distribution of nucleocytoplasmic fluorescence of ANXA1 in (C). **(E)** Tat-NTS peptide dose-dependently altered ANXA1 protein levels in the cytoplasm and nucleus. Primary cultured microglia subjected to OGD/R were treated with increasing amounts of Tat-NTS. **(F)** Quantification analysis of the Western blots shown in (E). **(G)** Tat-NTS peptide altered ANXA1 protein levels in the cytoplasm and nucleus in a time-dependent manner, Tat-NTS peptide or PBS treatments were removed at different time points after OGD/R. **(H and I)** Quantification of the Western blots shown in (G). **(J and K)** Collection of supernatant from primary cultured microglia in the indicated group. LDH assay indicating LDH release (J) and cell viability was detected by CCK-8 assay (K). **(L)** Schematic drawing corresponding to animal experiments in Figure 1, Figure 2, Figure 3A-B, and Figure S3. Data are presented as mean ± SEM and analyzed by two-way ANOVA with Tukey's post hoc test. $p < 0.05$, **$p < 0.01$, ***$p < 0.001$, and ****$p < 0.0001$, ns, not significant ($p > 0.05$).

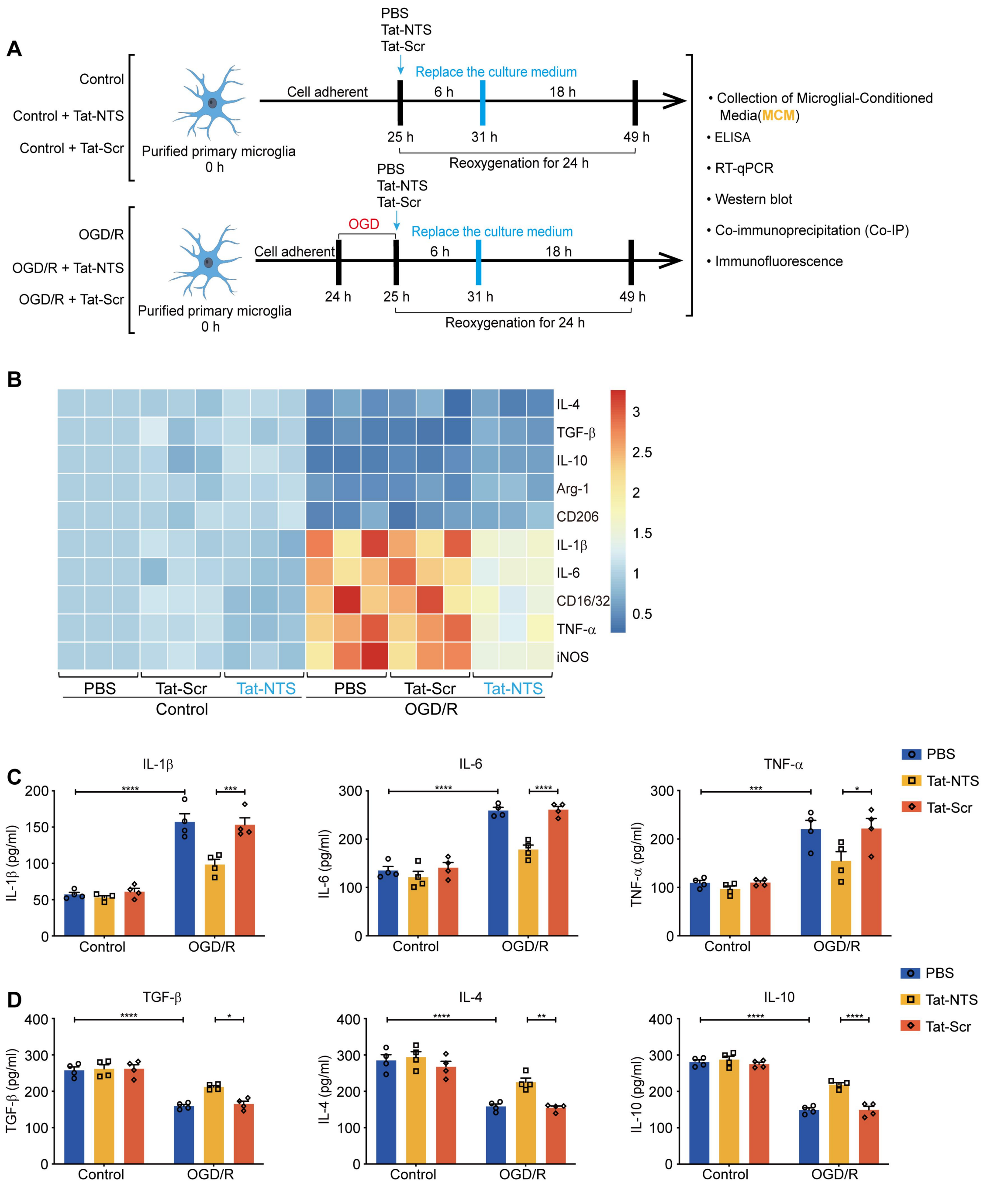

Figure S2. Tat-NTS peptide inhibits the expression of pro-inflammatory cytokines and upregulates the expression of anti-inflammatory cytokines in microglia after OGD/R.

(A) Schematic diagram corresponding to in vitro experiments for primary cultured microglia in the indicated group. **(B)** Heat map generated by R language showing the average mRNA expression in Figure 2A and B using a color scale from blue (downregulated) to red (upregulated). **(C and D)** Expression levels of pro-inflammatory cytokines IL-1β, IL-6, and TNF-α (C) and anti-inflammatory cytokines TGF-β, IL-4, and IL-10 (D) from microglial supernatants in the indicated group of (A) were

measured by ELISA. Data are presented as mean ± SEM and analyzed by two-way ANOVA with Tukey's post hoc test. *$p < 0.05$, **$p < 0.01$, ***$p < 0.001$, and ****$p < 0.0001$.

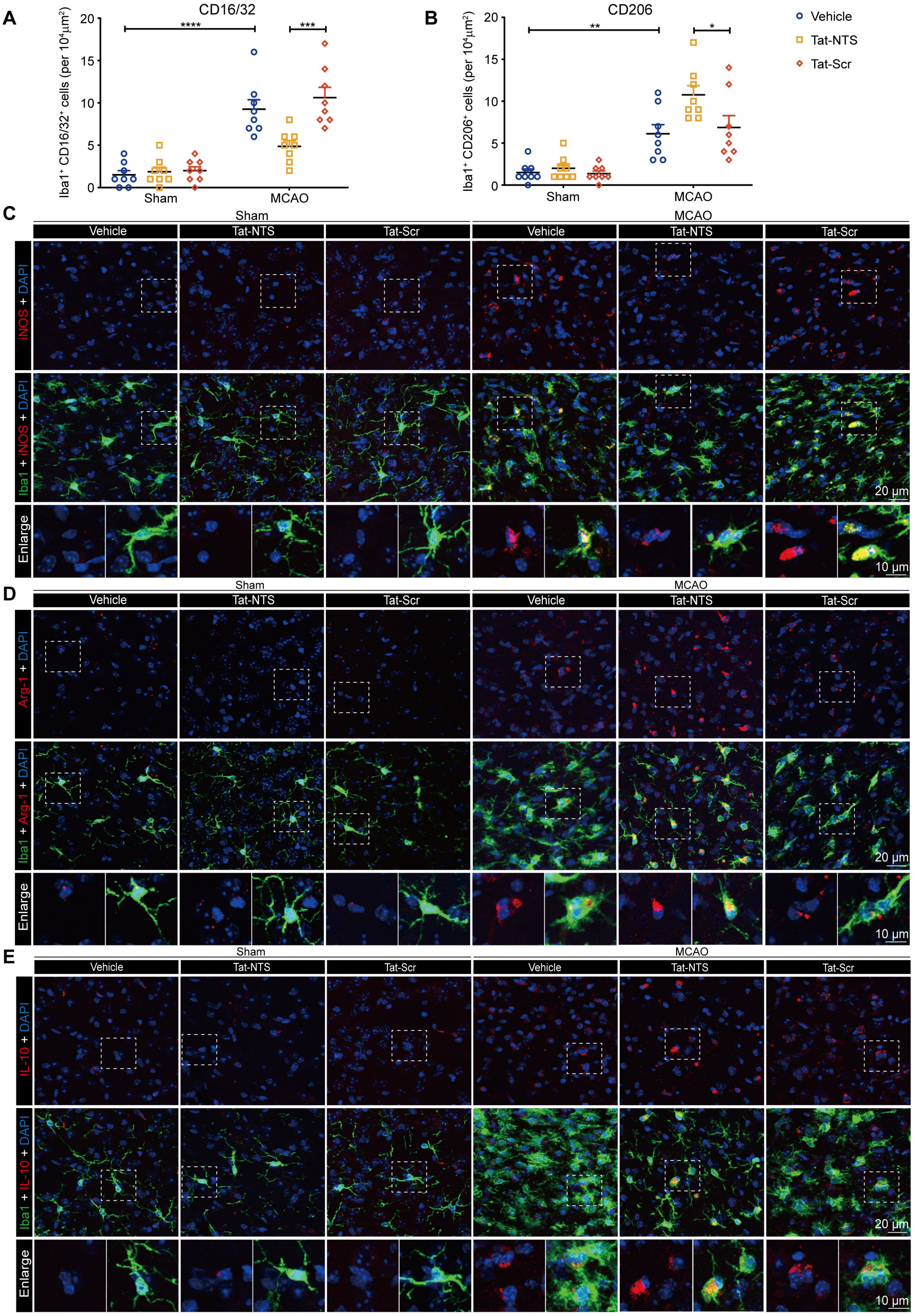

Figure S3. Tat-NTS peptide induces microglial polarization after ischemic injury to switch to the reparative anti-inflammatory phenotype.

(A) Quantitative analysis of the number of CD16/32⁺ Iba1⁺ cells per 10^4 μm^2. **(B)** Quantitative analysis

of the number of $CD206^+$ $Iba1^+$ cells per 10^4 μm^2. Each data point in (A) and (B) represents the number of cells from a single field of view, 8 randomly selected fields of view from n = 4 mice. **(C)** Representative immunofluorescence images of triple labeling of iNOS (red), Iba1 (green) and DAPI (blue), **(D)** Representative immunofluorescence images of triple labeling of Arg-1 (red), Iba1 (green) and DAPI (blue) and **(E)** Representative immunofluorescence images of triple labeling of IL-10 (red), Iba1 (green) and DAPI (blue) from the ischemic penumbra of brain tissue of wild-type mice; row 3, enlargement images of areas of interest indicated in row 1 and 2 by white dashed box; the scale bar of row 1-2, 20 μm; the scale bar of row 3, 10 μm. Data are presented as mean ± SEM and analyzed by two-way ANOVA with Tukey's post hoc test. $^*p < 0.05$, $^{**}p < 0.01$, $^{***}p < 0.001$, and $^{****}p < 0.0001$.

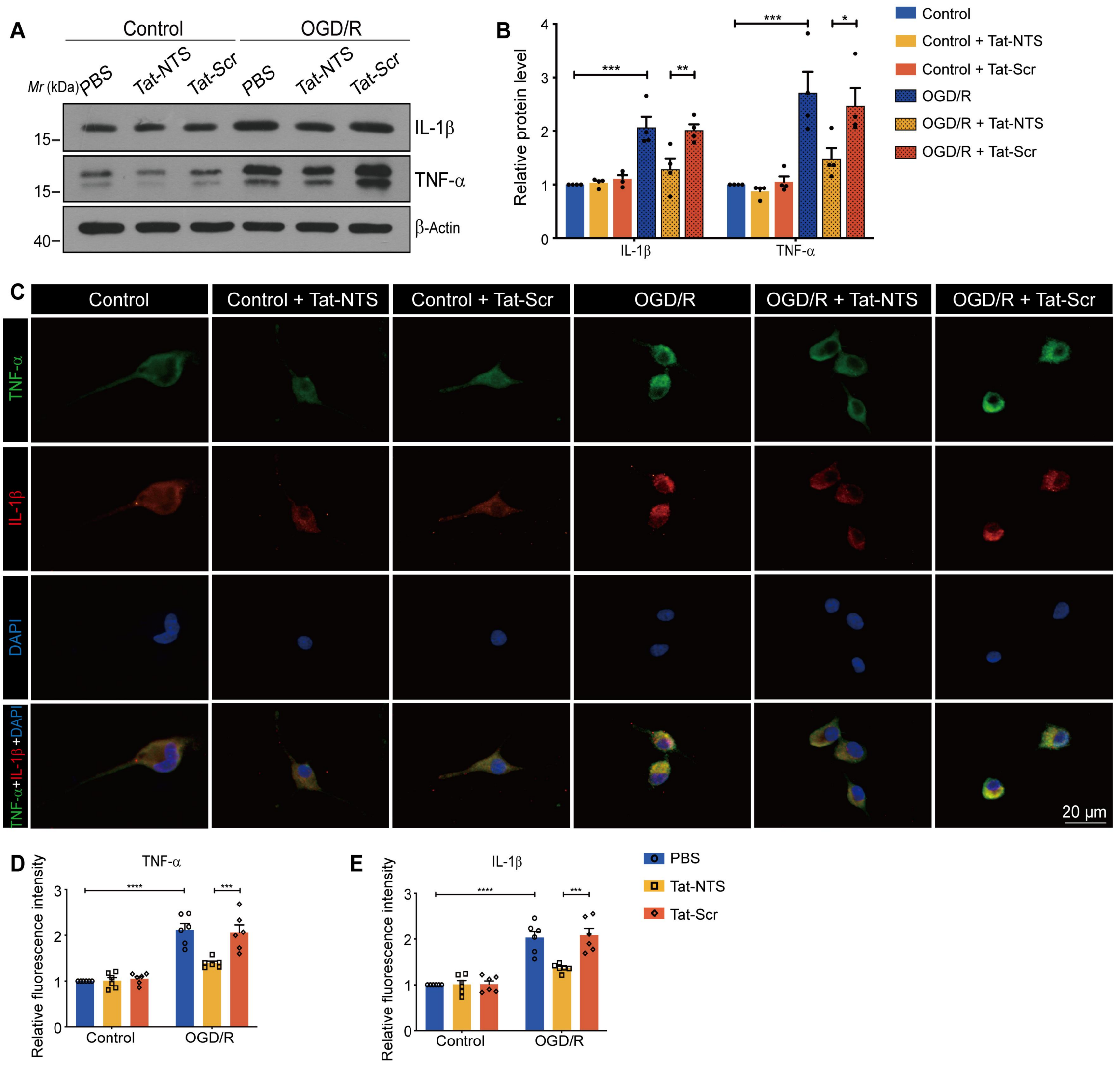

Figure S4. Tat-NTS peptide decreased the expression of classic inflammatory markers IL-1β and TNF-α in OGD/R-treated microglia.

(A) Representative images of immunoblot analysis of IL-1β and TNF-α in primary cultured microglia. **(B)** Quantitative analysis of the data shown in (A). **(C)** Immunofluorescence staining of TNF-α (green) and IL-1β (red) co-labeled with DAPI (blue) in primary cultured microglia. Scale bar, 20 μm. **(D and E)** Fluorescence intensity of TNF-α (D) and IL-1β (E) was quantified using ImageJ software (FIJI). Data are presented as means ± SEM and analyzed by two-way ANOVA followed by Tukey's post hoc test. *$p < 0.05$, **$p < 0.01$, ***$p < 0.001$, and ****$p < 0.0001$.

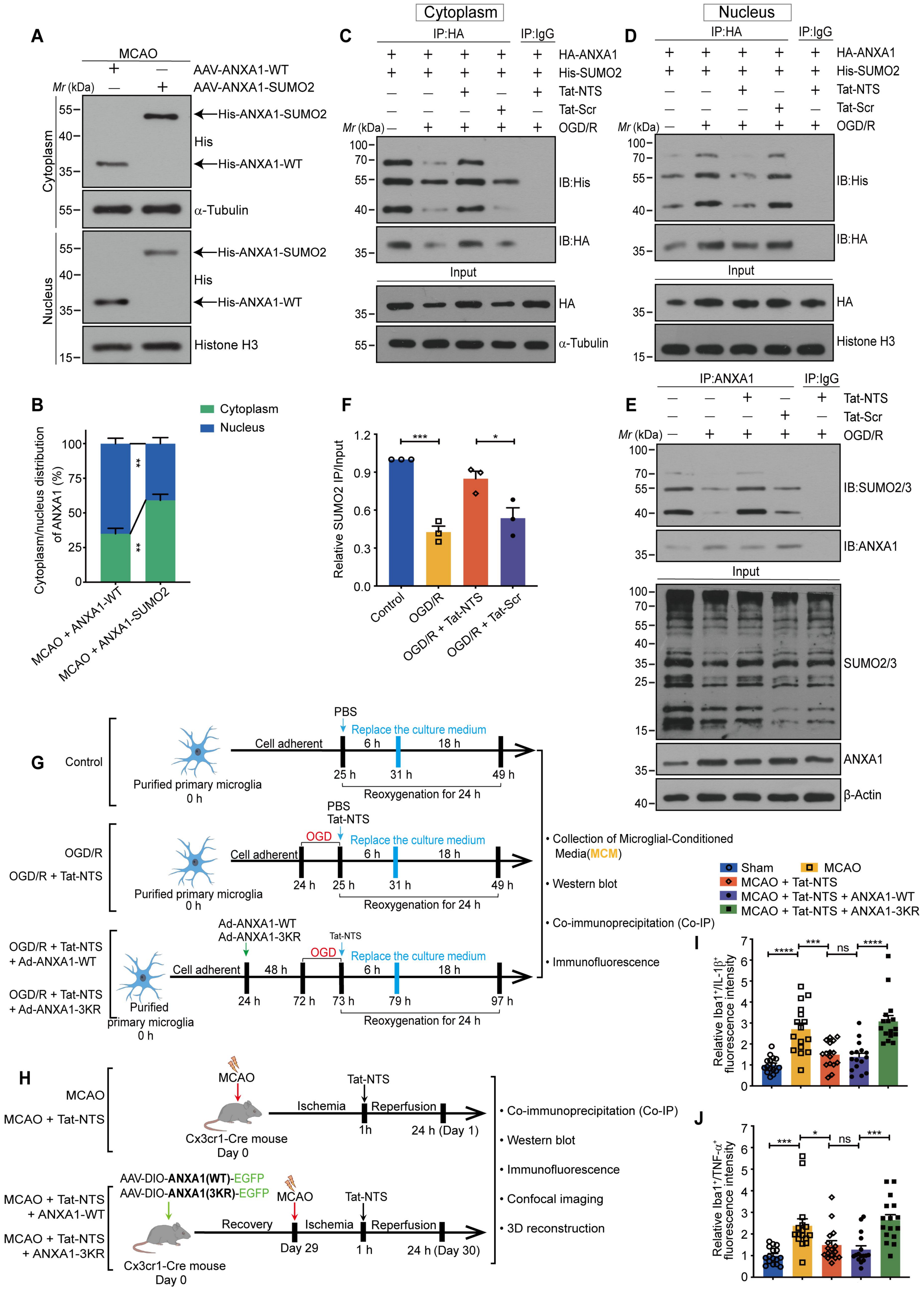

Figure S5. Tat-NTS peptide upregulates the SUMOylation levels of ANXA1 in the cytoplasm after ischemic injury and suppresses the expression of ischemic injury-induced pro-inflammatory factor IL-1β and TNF-α by ANXA1 SUMOylation.

(A) Representative immunoblotting for protein expression of ANXA1 or ANXA-SUMO2 in cytoplasmic extracts, nuclear extracts in adult male Cx3cr1-Cre mice using His-tag antibodies, the structure of the adeno-associated viruses (AAVs) shown in (A) refer to Figure S9A. **(B)** Quantification analysis of the Western blots shown in (A)**. (C and D)** HEK293T cells were co-transfected with His-SUMO2 and HA-ANXA1, cytoplasmic and nuclear proteins were isolated separately and then used for IP experiment to detect the interaction between ANXA1 and SUMO2 in the cytoplasm (C) and nucleus (D) under the treatment of Tat-NTS peptide. **(E)** Tat-NTS peptide upregulates OGD/R-mediated decrease in binding between ANXA1 and SUMO2 in microglia. IgG, immunoglobulin G. **(F)** Quantitative analysis of SUMO2 interaction with ANXA1 in the data shown in (E). **(G)** Experimental schedule and schematic diagram of the operation for primary cultured microglial cells in the indicated groups. **(H)** Timeline of virus injection and the operation for Cx3cr1-Cre mice from indicated groups. **(I and J)** Quantification of the IL-1β^+ (I) or TNF-α^+ (J) fluorescence intensity in Iba1$^+$ cells by Fiji ImageJ. Each data point represents the average fluorescence intensity of all positive cells from a single field of view, 16 randomly selected fields of view from n = 4 mice. Data are presented as mean ± SEM and analyzed by one-way ANOVA (F, I and J) and two-way ANOVA (B) with Tukey's post hoc test. ns, not significant ($p > 0.05$), *$p < 0.05$, **$p < 0.01$, ***$p < 0.001$, and ****$p < 0.0001$.

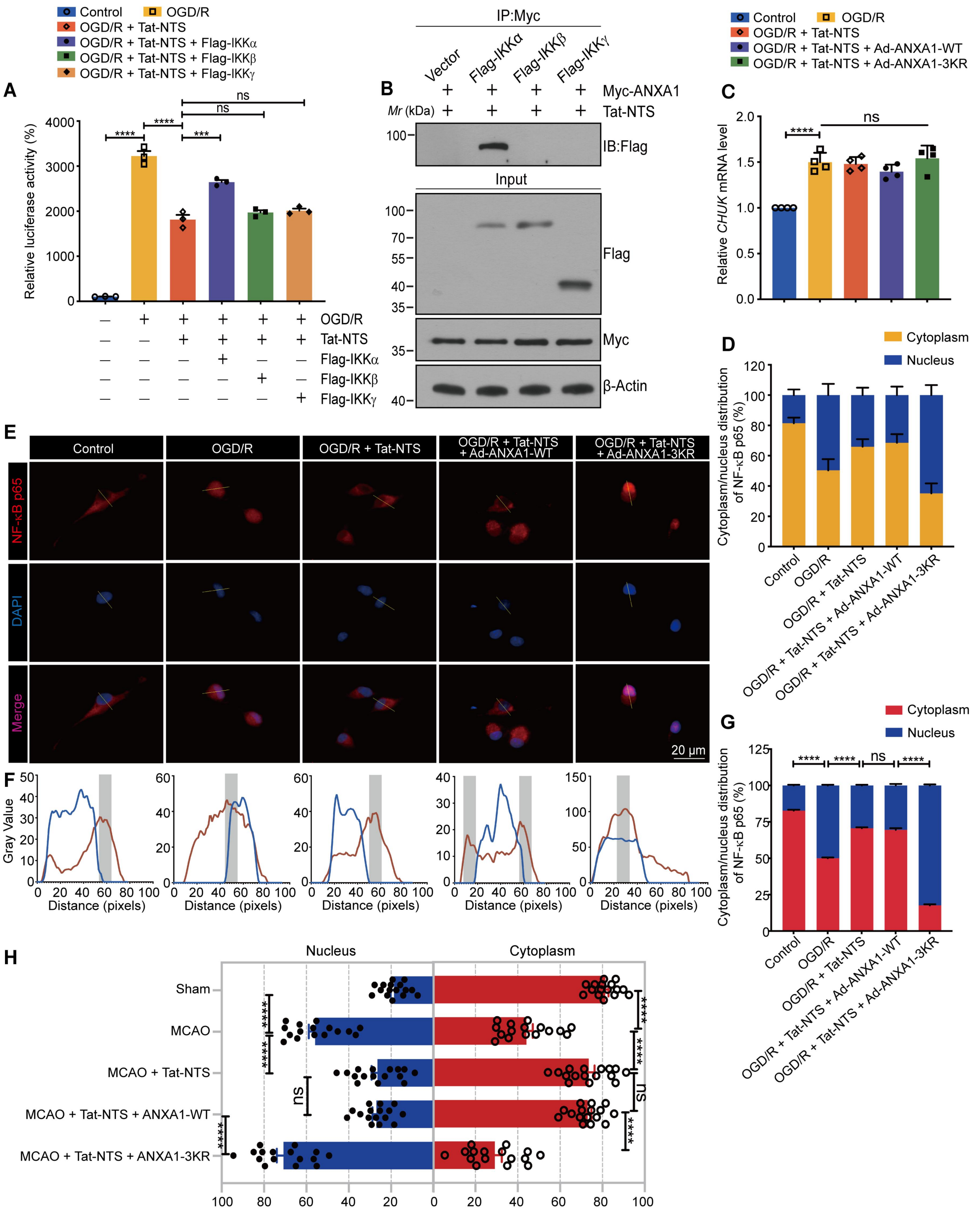

Figure S6. Tat-NTS peptide enhances IKKα degradation and blocks nuclear translocation of NF-κB p65 after ischemic injury.

(A) The transcriptional activity of NF-κB p65 upon treatment with Tat-NTS peptide and overexpression of different IKK subunits was measured using a dual luciferase reporter assay. **(B)** ANXA1 was bound to IKKα in HEK293T cells by immunoprecipitation with Myc-tag antibody. **(C)** *CHUK* mRNA expression was analyzed by qRT-PCR. **(D)** The nucleocytoplasmic protein percentage distribution of

NF-κB p65 in Figure 4F and G. **(E)** The immunofluorescence images for NF-κB p65 (red) and DAPI (blue) in OGD/R-stimulated microglia under Tat-NTS treatment. Scale bar, 20 μm. **(F)** Merged profiles of fluorescence intensity of NF-κB p65 (red line) and DAPI (blue line) signals along the yellow lines crossing cells as shown. Light gray areas indicate the NF-κB p65 peak. **(G)** Quantitative analysis of the percentage distribution of nucleocytoplasmic fluorescence of NF-κB p65 in (J) ($n = 50$ cells). **(H)** Quantitative analysis of the percentage of nucleocytoplasmic fluorescence distribution of NF-κB p65 using Fiji ImageJ software, each data point represents the average of all microglia in a single field of view, 16 randomly selected fields of view from $n = 4$ mice. Data are presented as mean ± SEM and analyzed by one-way ANOVA (A, C and H) and two-way ANOVA (G) with Tukey's post hoc test. ns, not significant ($p > 0.05$), ***$p < 0.001$, and ****$p < 0.0001$.

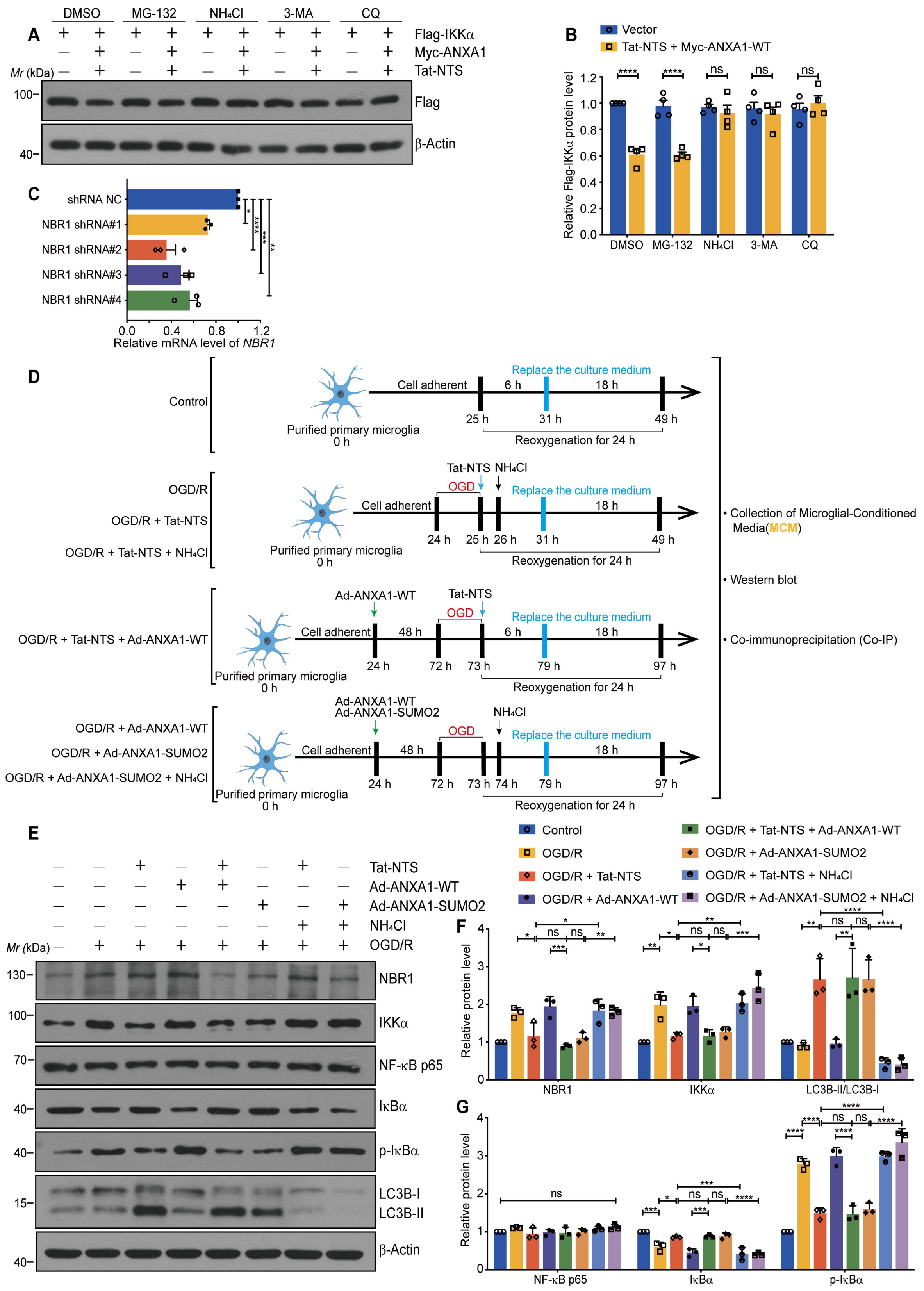

Figure S7. Tat-NTS peptide enhances IKK α degradation through NBR1-dependent selective autophagy.

(A and B) Immunoblotting analysis measured the expression level of Flag-IKKα in Tat-NTS

peptide-treated primary cultured microglia with Myc-ANXA1 overexpressing under administration of different inhibitors (A) and quantification analysis (B). **(C)** qRT-PCR quantitative analysis of *NBR1* mRNA in HEK293T cells transfected with different NBR1 shRNAs. **(D)** Experimental schedule and schematic diagram of the operation for primary cultured microglial cells in the indicated groups. **(E-G)** Immunoblotting analysis measured the expression level of indicated proteins in different AAVs infected and/or Tat-NTS peptide treated primary cultured microglia (E) and quantification analysis (F, G). Data are presented as mean ± SEM and analyzed by one-way ANOVA (C, F and G) and two-way ANOVA (B) with Tukey's post hoc test. ns, not significant ($p > 0.05$), *$p < 0.05$, **$p < 0.01$, ***$p < 0.001$, and ****$p < 0.0001$.

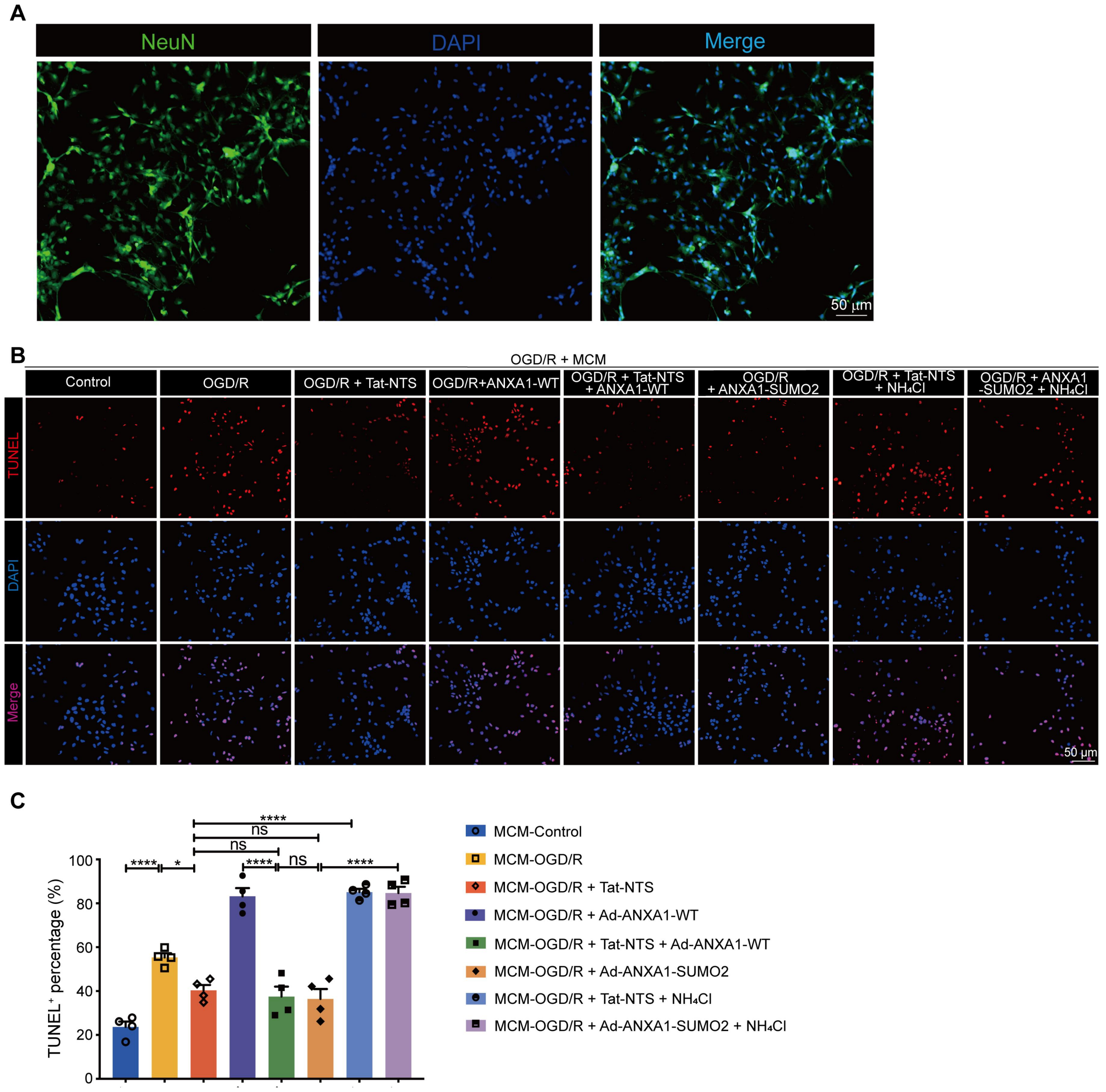

Figure S8. Microglia treated with Tat-NTS peptide improves neuronal survival under OGD/R conditions.

(A) Purity of primary cultured neuronal cells was measured by immunofluorescence analysis. Cells were stained with the neuron-specific marker NeuN (green). Scale bar, 50 μm. **(B and C)** Representative images of TUNEL staining (B) and quantitative analysis (C) showing that the MCM from Tat-NTS peptide-treated microglia protects neurons against neuronal apoptosis after OGD/R, which is dependent on ANXA1 SUMOylation in microglia. Scale bar, 50 μm. Data in (C) are presented as the mean ± SEM and analyzed by one-way ANOVA with Tukey's post hoc test. ns, not significant ($p > 0.05$), *$p < 0.05$ and ****$p < 0.0001$.

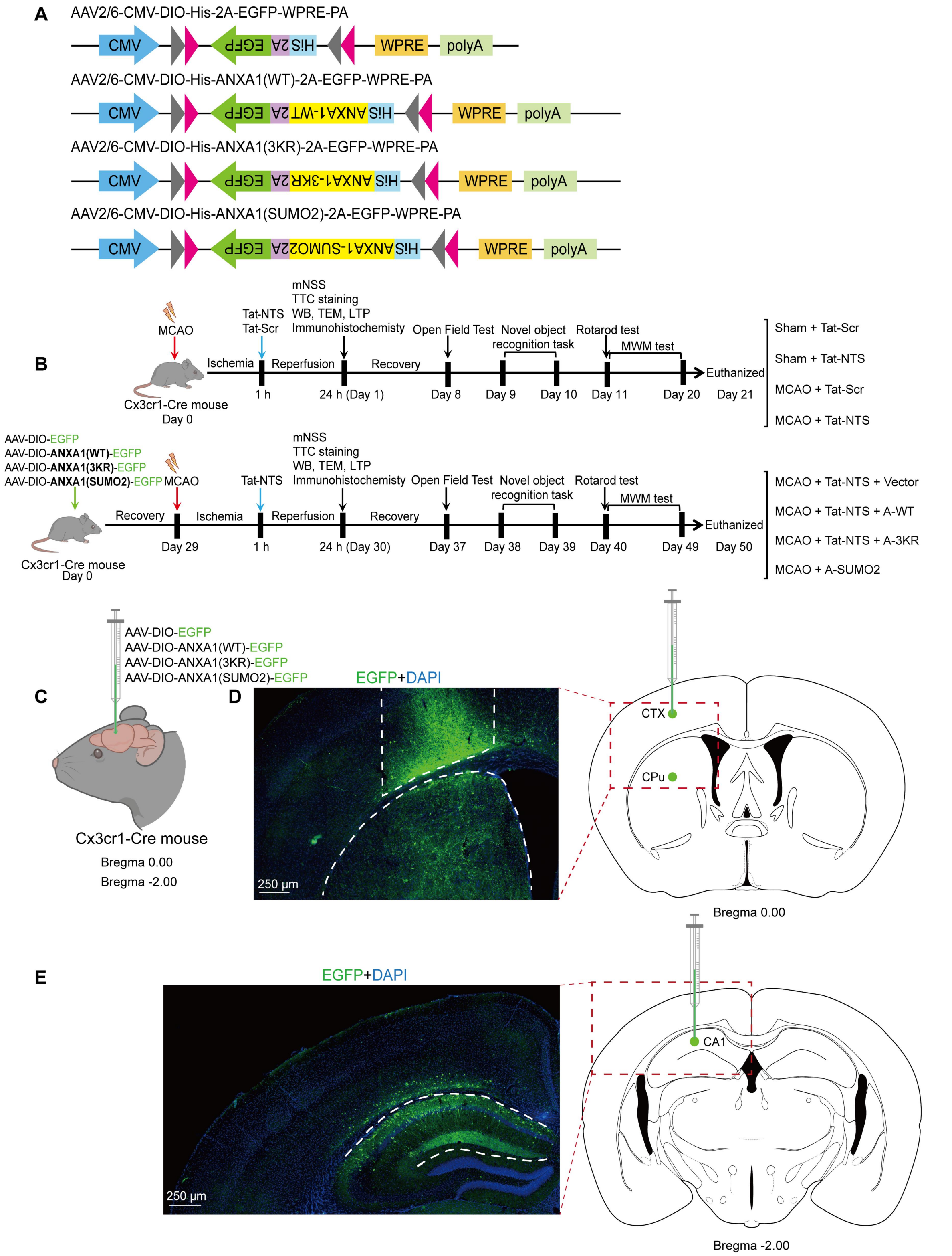

Figure S9. AAVs transfection manipulate gene overexpression in microglia/macrophages from specific regions of mice.

(A) Schematic of AAVs vectors for microglia/macrophages overexpressing ANXA1-WT, ANXA1-3KR or ANXA1-SUMO2. DIO, double-flexed inverted open reading frame; EGFP, enhanced GFP; ITR,

inverted terminal repeat; WPRE, woodchuck hepatitis virus post-transcriptional regulatory element. **(B)** Timeline of virus injection and in vivo experimental procedure for Cx3cr1-Cre mice from the indicated groups. **(C-E)** Schematic diagram of different virus injections into different brain regions (Bregma anterior-posterior: 0.00 mm for cortex and corpus striatum, Bregma anterior-posterior: -2.00 mm for hippocampus) (C) and representative image from virus-injected mice (D, E). Scale bar, 250 μm.

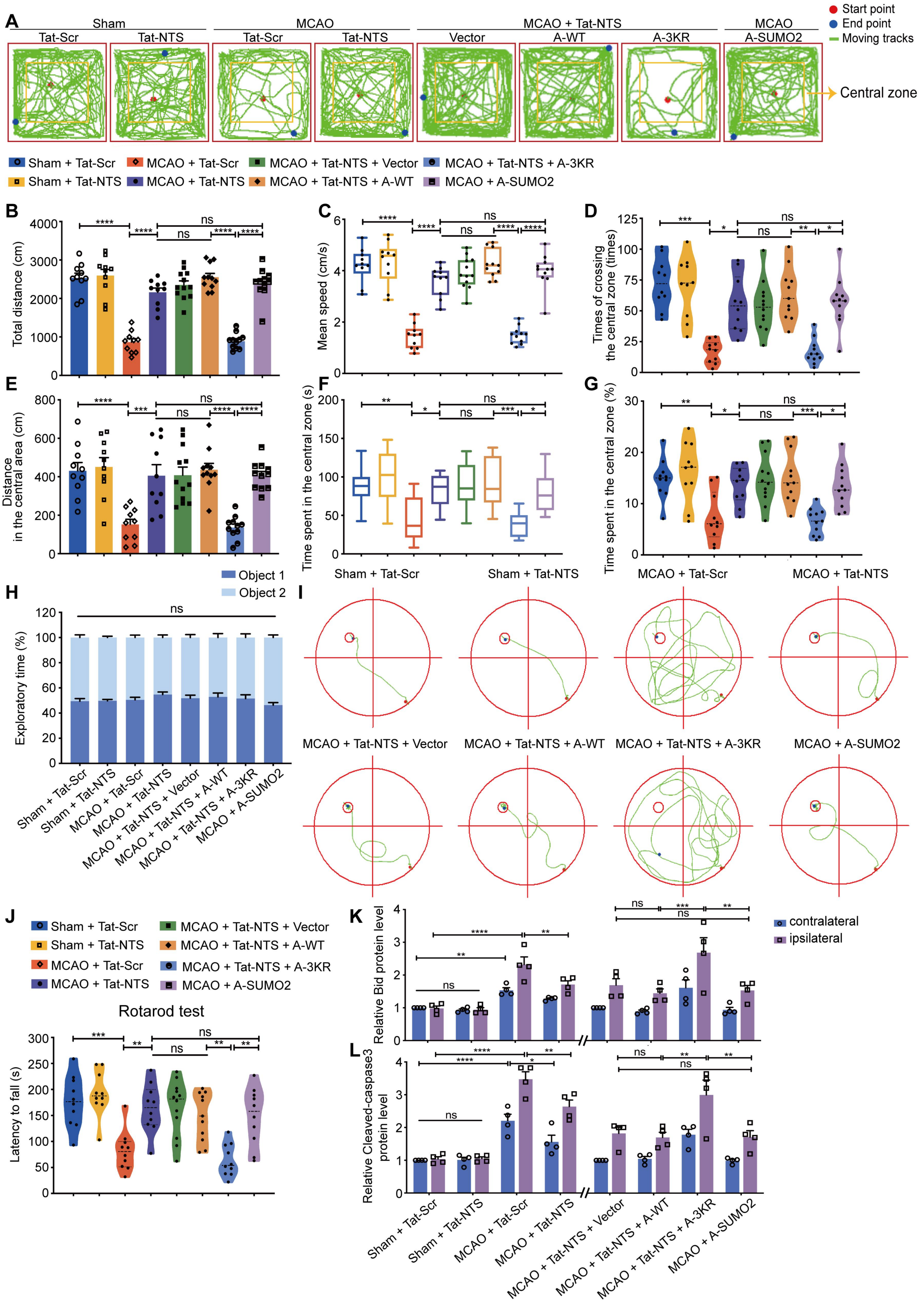

Figure S10. Tat-NTS peptide-treated mice show improved motor and cognitive function, and inhibited apoptosis levels after ischemic cerebral injury.

(A) Representative track traces of each respective group in the open field test (OFT). **(B-G)** Quantitative analysis of total distance (B), average speed (C), central zone crossing times (D), central zone distance

(E), time spent in the central zone (F), and percentage of time spent in the central zone (G). **(H)** Percentage of time spent exploring two identical familiar objects in the training session. **(I)** The representative swim path showing sample paths of mice from training trials on day 7. **(J)** The time spent in the rotarod test of mice from different groups. **(K and L)** Quantitative analysis of the protein expression of apoptotic factors Bid (K) and cleaved-caspase3 (L) in Figure 7R. Data are presented as means ± SEM and analyzed by one-way ANOVA (B-G and J) or two-way ANOVA (H, K and L) with Tukey's post hoc test or Dunnett's post hoc test (D, J). ns, not significant ($p > 0.05$), *$p < 0.05$, **$p < 0.01$, ***$p < 0.001$, and ****$p < 0.0001$. (n = 10-12 mice per group).

Table S1. Antibodies used in this study.

Antibody	Species	Type	IB	IF	Source	Identifier
ANXA1	Mouse	Mono-	1:1000	1:100	Proteintech	66344-1-Ig
ANXA1	Rabbit	Poly-	1:1000	1:200	Proteintech	21990-1-AP
β-actin	Mouse	Mono-	1:1000		Santa Cruz	sc-47778
Iba1	Rabbit	Poly-		1:500	Wako	#019-19741
Iba1	Goat	poly		1:100	Abcam	ab5076
iNOS	Rabbit	Poly-	1:500	1:100	Proteintech	18985-1-AP
Arg-1	Rabbit	Mono-	1:1000	1:200	Cell Signaling	#93668
CD16/32	Rat	Mono-	1:500	1:200	BD Biosciences	#553141
CD206	Goat	Poly-		1:200	R&D Systems	AF2535
CD206	Rabbit	Poly-	1:1000		Immunoway	YT5640
IL-1β	Rabbit	Poly-	1:1000	1:200	Bioss	bs-0812R
TNF-α	Rabbit	Poly-	1:1000	1:200	Immunoway	YT4689
IL-10	Mouse	Mono-		1:100	Proteintech	60269-1-1g
HA	Mouse	Mono-	1:1000		Santa Cruz	sc-7392
His	Rabbit	Poly-	1:1000		Sigma-Aldrich	SAB1306085
SUMO-2/3	Rabbit	Mono-	1:1000		Cell Signaling	#4971
IκBα	Mouse	Mono-	1:1000		Cell Signaling	#4814
Phospho-IκBα	Rabbit	Mono-	1:1000		Cell Signaling	#2859
IKKα	Mouse	Mono-	1:1000	1:200	Cell Signaling	#11930
IKKβ	Rabbit	Mono-	1:1000		Cell Signaling	#8943
IKKγ	Mouse	Mono-	1:1000		Santa Cruz	sc-8032
Myc	Mouse	Mono-	1:3000		Abmart	M20002
NF-κB p65	Rabbit	Mono-	1:1000	1:200	Cell Signaling	#8242
α-tubulin	Mouse	Mono-	1:2000		Santa Cruz	sc-8035
Histone H3	Rabbit	Mono-	1:2000		Cell Signaling	#4499
LC3A/B	Rabbit	Poly-	1:1000		Cell Signaling	#12741
LAMP2A	Rabbit	Mono-	1:1000		Abcam	ab125068
Flag	Mouse	Mono-	1:2000		Santa Cruz	sc-166355
NBR1	Rabbit	Mono-	1:1000		Proteintech	16004-1-AP
NeuN	Rabbit	Mono-		1:200	Abcam	EPR12763
BID	Rabbit	Poly-	1:1000		Immunoway	YT0488
Cleaved-caspase 3	Rabbit	Poly-	1:1000		Proteintech	19677-1-AP

Abbreviations: IB, immunoblotting; IF, immunofluorescence.

Table S2. Primers used in this study.

Primer name	Primer sequences (5'-3')	
	**Forward**	**Reverse**
**Quantitative RT-PCR primers**		
IL-1β	GAAAGACGGCACACCCAC	TGTGACCCTGAGCGACCT
IL-6	TCTCTGGGAAATCGTGGAA	GATGGTCTTGGTCCTTAGCC
TNF-α	ACGGCATGGATCTCAAAGAC	AGATAGCAAATCGGCTGACG
iNOS	GCTTGTCTCTGGGTCCTCTG	CTCACTGGGACAGCACAGAA
CD16/32	ACAACCCTGGGAACTCTTCTAC	GGTTGGCTTTTGGGATAGA
Arg-1	CAAGACAGGGCTCCTTTCAG	TGGCTTATGGTTACCCTCCC
IL-4	CCCCCAGCTAGTTGTCATCC	AGGACGTTTGGCACATCCAT
IL-10	CTGCCTGCTCTTACTGACTG	AAATCACTCTTCACCTGCTC
TGF-β	TGCGCTTGCAGAGATTAAAA	CGTCAAAAGACAGCCACTCA
CD206	TCAGCTATTGGACGCGAGGCA	TCCGGGTTGCAAGTTGCCGT
NBR1	CCAGAGGCTCATCAGGACTTGTA	CAAGGTCACTCCTCAATAGCGTT
CHUK	GACTTGATGGAATCTCTGGA	GATGCCATATTTCTTTCTGC
β-ACTIN	GGCTGTATTCCCCTCCATCG	CCAGTTGGTAACAATGCCATGT
**Genotyping primers**		
Cx3cr1 Cre	CAACGAGTGATGAGGTTCGCAAG	ACACCAGAGACGGAAATCCATCG

Table S3. Statistical analyses for all figures.

Figure Number	n	Primary statistic	Post-hoc test	P value	Degrees of Freedom & F Value
**1D**	4 mice per group, 16 fields of view	Two-way ANOVA	Tukey's post hoc test	$P < 0.0001$	$F_{5,75} = 223.5$
**1E**	4 mice per group, 16 fields of view	Two-way ANOVA	Tukey's post hoc test	$P < 0.0001$	$F_{1,90} = 109.5$
**2A**	3 or 4 per group	Two-way ANOVA	Tukey's post hoc test	*IL-1β*, $P < 0.0001$	$F_{1,18} = 156.8$
				IL-6, $P < 0.0001$	$F_{1,12} = 143.6$
				TNF-α, $P < 0.0001$	$F_{1,18} = 238.1$
				iNOS, $P < 0.0001$	$F_{1,12} = 78.43$
				CD16/32, $P < 0.0001$	$F_{1,12} = 72.71$
**2B**	3 per group	Two-way ANOVA	Tukey's post hoc test	*Arg-1*, $P < 0.0001$	$F_{1,12} = 148.2$
				TGF-β, $P < 0.0001$	$F_{1,12} = 108.9$
				IL-4, $P < 0.0001$	$F_{1,12} = 95.27$
				IL-10, $P < 0.0001$	$F_{1,12} = 151.2$
				CD206, $P < 0.0001$	$F_{1,12} = 102.2$
**2D**	3 per group	Two-way ANOVA	Tukey's post hoc test	iNOS, $P < 0.0001$	$F_{1,12} = 39.90$
				Arg-1, $P < 0.0001$	$F_{1,12} = 78.19$
				CD16/32, $P < 0.0001$	$F_{1,12} = 417.8$
				CD206, $P < 0.0001$	$F_{1,12} = 508.2$
**3B**	3 mice per group	Two-way ANOVA	Tukey's post hoc test	$P < 0.0001$	$F_{1,8} = 180.7$
**3C**	3 per group	One-way ANOVA	Tukey's post hoc test	$P < 0.0001$	$F_{4,10} = 409.7$
**3E**	3 per group	One-way ANOVA	Tukey's post hoc test	IκBα, $P < 0.0001$	$F_{4,10} = 23.82$
				p-IκBα, $P = 0.0008$	$F_{4,10} = 11.87$
**3G**	3 per group	One-way ANOVA	Tukey's post hoc test	IL-1β, $P = 0.0005$	$F_{4,10} = 13.13$
				TNF-α, $P = 0.0001$	$F_{4,10} = 18.51$
**4B**	3 per group	One-way ANOVA	Tukey's post hoc test	IKKα, $P < 0.0001$	$F_{4,10} = 20.46$
				IKKβ, $P < 0.0001$	$F_{4,10} = 29.87$
				IKKγ, $P = 0.2308$	$F_{4,10} = 1.678$
**4E**	5 per group	Two-way repeated measures (RM) ANOVA	Tukey's post hoc test	$P < 0.0001$	$F_{2,48} = 13.52$
**4G**	3 per group	One-way ANOVA	Tukey's post hoc test	Whole-cell lysates, $P = 0.6736$	$F_{4,10} = 0.5962$
				Cytoplasm, $P <$	$F_{4,10} = 61.35$

				0.0001	
				Nucleus, $P = 0.0003$	$F_{4,10} = 15.24$
**5B**	4 mice per group, 16 fields of view	One-way ANOVA	Tukey's post hoc test	$P < 0.0001$	$F_{4,75} = 42.40$
**5D**	3 per group	Two-way ANOVA	Šídák's multiple comparisons test	$P = 0.0011$	$F_{1,20} = 14.41$
**5F**	3 per group	One-way ANOVA	Tukey's post hoc test	$P < 0.0001$	$F_{5,12} = 26.92$
**5H**	3 per group	One-way ANOVA	Tukey's post hoc test	Lysosomes, $P < 0.0001$	$F_{5,12} = 27.98$
				Homogenates, $P < 0.0001$	$F_{5,12} = 16.60$
**5J**	5 per group	One-way ANOVA	Tukey's post hoc test	$P < 0.0001$	$F_{4,20} = 29.68$
**5L**	3 per group	One-way ANOVA	Tukey's post hoc test	IKKα, $P < 0.0001$	$F_{5,12} = 16.51$
				LC3B-II/I, $P = 0.0012$	$F_{5,12} = 8.564$
**5M**	3 per group	One-way ANOVA	Tukey's post hoc test	NF-κB p65, $P = 0.7746$	$F_{5,12} = 0.4944$
				IκBα, $P < 0.0001$	$F_{5,12} = 26.84$
				p-IκBα, $P < 0.0001$	$F_{5,12} = 171.5$
**6C**	3 per group	One-way ANOVA	Tukey's post hoc test	Bid, $P < 0.0001$	$F_{5,12} = 42.78$
				Cleaved-caspase3, $P = 0.0004$	$F_{5,12} = 10.96$
**6E**	3 per group	One-way ANOVA	Tukey's post hoc test	Bid, $P < 0.0001$	$F_{5,12} = 25.74$
				Cleaved-caspase3, $P < 0.0001$	$F_{5,12} = 16.31$
**6G**	3 per group	One-way ANOVA	Tukey's post hoc test	Bid, $P < 0.0001$	$F_{7,16} = 13.49$
				Cleaved-caspase3, $P < 0.0001$	$F_{7,16} = 16.00$
**6I**	4 per group	Two-way ANOVA	Tukey's post hoc test	$P < 0.0001$	$F_{1,18} = 161.0$
**6K**	4 per group	One-way ANOVA	Tukey's post hoc test	$P < 0.0001$	$F_{5,18} = 43.44$
**7B**	6 mice per group	One-way ANOVA	Tukey's post hoc test	$P < 0.0001$	$F_{7,40} = 87.43$
**7C**	10-12mice per group	Kruskal-Wallis non-parametric test	Dunnett's post hoc test	$P < 0.0001$	

**7E**	10-12 mice per group	Two-way ANOVA	Tukey's post hoc test	$P < 0.0001$	$F_{1,154} = 14.86$
**7G**	10-12mice per group	Two-way ANOVA	Tukey's post hoc test	$P < 0.0001$	$F_{42,539} = 2.270$
**7H**	10-12mice per group	One-way ANOVA	Tukey's post hoc test	$P < 0.0001$	$F_{7,77} = 5.397$
**7I**	10-12mice per group	One-way ANOVA	Tukey's post hoc test	$P < 0.0001$	$F_{7,77} = 5.466$
**7J**	10-12mice per group	Kruskal-Wallis non-parametric test	Dunnett's post hoc test	$P = 0.0001$	
**7K**	3 mice, 6 slices per group	Two-way ANOVA	Tukey's post hoc test	$P < 0.0001$	$F_{180,7124} = 198.5$
**7L**	3 mice, 6 slices per group	One-way ANOVA	Tukey's post hoc test	$P < 0.0001$	$F_{7,40} = 32.03$
**7N**	3 mice, 6 slices per group	Kruskal-Wallis non-parametric test	Dunnett's post hoc test	$P < 0.0001$	
**7P**	4 mice per group	Two-way ANOVA	Tukey's post hoc test	$P < 0.0001$	$F_{1,48} = 66.10$
**7Q**	4 mice per group	Two-way ANOVA	Tukey's post hoc test	$P < 0.0001$	$F_{1,48} = 62.93$
**S1A**	4 mice per group, 18 fields of view	Two-way ANOVA	Tukey's post hoc test	$P < 0.0001$	$F_{1,68} = 520.7$
**S1D**	50 cells per group	Two-way ANOVA	Tukey's post hoc test	$P < 0.0001$	$F_{1,196} = 545.1$
**S1F**	3 per group	Two-way ANOVA	Tukey's post hoc test	$P < 0.0001$	$F_{5,24} = 91.52$
**S1H**	3 per group	Two-way ANOVA	Tukey's post hoc test	$P = 0.0668$	$F_{5,24} = 2.402$
**S1I**	3 per group	Two-way ANOVA	Tukey's post hoc test	$P < 0.0001$	$F_{5,24} = 22.85$
**S1J**	3 per group	Two-way ANOVA	Tukey's post hoc test	$P < 0.0001$	$F_{2,12} = 34.96$
**S1K**	3 per group	Two-way ANOVA	Tukey's post hoc test	$P < 0.0001$	$F_{1,12} = 148.7$
**S2C**	4 per group	Two-way ANOVA	Tukey's post hoc test	IL-1β, $P < 0.0001$	$F_{1,18} = 185.1$
				IL-6, $P < 0.0001$	$F_{1,18} = 183.2$
				TNF-α, $P < 0.0001$	$F_{1,18} = 65.19$
**S2D**	4 per group	Two-way	Tukey's post	TGF-β, $P < 0.0001$	$F_{1,18} = 138.5$
				IL-4, $P < 0.0001$	$F_{1,18} = 106.0$

		ANOVA	hoc test	IL-10, $P < 0.0001$	$F_{1,18} = 354.5$
**S3A**	4 mice per group, 8 fields of view	Two-way ANOVA	Tukey's post hoc test	$P < 0.0001$	$F_{1,42} = 94.22$
**S3B**	4 mice per group, 8 fields of view	Two-way ANOVA	Tukey's post hoc test	$P < 0.0001$	$F_{1,42} = 72.52$
**S4B**	4 per group	Two-way ANOVA	Tukey's post hoc test	IL-1β, $P < 0.0001$	$F_{1,18} = 49.45$
				TNF-α, $P < 0.0001$	$F_{1,18} = 46.60$
**S4D**	6 per group	Two-way ANOVA	Tukey's post hoc test	$P < 0.0001$	$F_{1,30} = 121.3$
**S4E**	6 per group	Two-way ANOVA	Tukey's post hoc test	$P < 0.0001$	$F_{1,30} = 112.7$
**S5B**	3 per group	Two-way ANOVA	Tukey's post hoc test	$P = 0.0004$	$F_{1,8} = 33.76$
**S5F**	3 per group	One-way ANOVA	Tukey's post hoc test	$P = 0.0003$	$F_{3,8} = 22.77$
**S5I**	4 mice per group, 16 fields of view	Two-way ANOVA	Tukey's post hoc test	$P < 0.0001$	$F_{4,75} = 94.22$
**S5J**	4 mice per group, 16 fields of view	Two-way ANOVA	Tukey's post hoc test	$P < 0.0001$	$F_{4,75} = 10.41$
**S6A**	3 per group	Two-way ANOVA	Tukey's post hoc test	$P < 0.0001$	$F_{5,10} = 201.4$
**S6C**	4 per group	One-way ANOVA	Tukey's post hoc test	$P < 0.0001$	$F_{4,15} = 23.61$
**S6G**	50 cells per group	Two-way ANOVA	Tukey's post hoc test	$P < 0.0001$	$F_{1,490} = 1144$
**S6H**	4 mice per group, 16 fields of view	One-way ANOVA	Tukey's post hoc test	$P < 0.0001$	$F_{4,75} = 76.80$
**S7B**	4 per group	Two-way ANOVA	Šídák's multiple comparisons test	$P < 0.0001$	$F_{4,30} = 12.07$
**S7C**	3 per group	One-way ANOVA	Tukey's post hoc test	$P = 0.0001$	$F_{4,10} = 18.52$
**S7F**	3 per group	One-way ANOVA	Tukey's post hoc test	NBR1, $P < 0.0001$	$F_{7,16} = 13.47$
				IKKα, $P < 0.0001$	$F_{7,16} = 15.70$
				LC3B-II/I, $P <$	$F_{7,16} = 20.53$

				0.0001	
**S7G**	3 per group	Two-way ANOVA	Tukey's post hoc test	NF-κB p65, P = 0.2555	$F_{7,16} = 1.444$
				IκBα, $P < 0.0001$	$F_{7,16} = 23.70$
				p-IκBα, $P < 0.0001$	$F_{7,16} = 65.17$
**S8C**	4 per group	One-way ANOVA	Tukey's post hoc test	$P < 0.0001$	$F_{7,24} = 63.07$
**S10B**	10-12mice per group	One-way ANOVA	Tukey's post hoc test	$P < 0.0001$	$F_{7,77} = 42.81$
**S10C**	10-12mice per group	One-way ANOVA	Tukey's post hoc test	$P < 0.0001$	$F_{7,77} = 42.81$
**S10D**	10-12mice per group	Kruskal-Wallis non-parametric test	Dunnett's post hoc test	$P < 0.0001$	
**S10E**	10-12mice per group	One-way ANOVA	Tukey's post hoc test	$P < 0.0001$	$F_{7,77} = 11.65$
**S10F**	10-12mice per group	One-way ANOVA	Tukey's post hoc test	$P < 0.0001$	$F_{7,77} = 7.136$
**S10G**	10-12mice per group	One-way ANOVA	Tukey's post hoc test	$P < 0.0001$	$F_{7,77} = 7.178$
**S10H**	10-12mice per group	Two-way ANOVA	Tukey's post hoc test	$P = 0.1318$	$F_{1,154} = 2.295$
**S10J**	10-12mice per group	Kruskal-Wallis non-parametric test	Dunnett's post hoc test	$P < 0.0001$	
**S10K**	4 mice per group	Two-way ANOVA	Tukey's post hoc test	$P < 0.0001$	$F_{1,48} = 38.71$
**S10L**	4 mice per group	Two-way ANOVA	Tukey's post hoc test	$P < 0.0001$	$F_{1,48} = 64.13$
